# The Abundance of α-Chain-Centric TCRs in the Mouse Repertoire of Primarily Activated Effectors and Reactivated Memory T Cells

**DOI:** 10.34133/csbj.0026

**Published:** 2026-04-08

**Authors:** Anastasiia Kalinina, Marina Kubekina, Nadezhda Persiyantseva, Alexandra Bruter, Ludmila Khromykh, Dmitry Kazansky

**Affiliations:** ^1^ N.N. Blokhin National Medical Research Center of Oncology, Ministry of Health of the Russian Federation, 115478 Moscow, Russia.; ^2^ Center for Precision Genome Editing and Genetic Technologies for Biomedicine, Institute of Gene Biology, Russian Academy of Sciences, 119334 Moscow, Russia.

## Abstract

Chain centricity is a feature of some T cell receptors (TCRs), in which one hemi-chain (α or β) dominates in antigen recognition. The exact proportion of chain-centric TCRs in the native repertoire is currently unknown, as is their inclusion in the repertoires of different functional T cell subsets. Discovering these main issues will elucidate the origin of chain-centric clonotypes and greatly improve their detection in bulk repertoires. In this work, the frequency and abundance of clonotypes with the dominant-active α-chain TCR (TCRα), i.e., α-chain-centric clonotypes, were identified in the mouse repertoires of primarily activated effectors and reactivated memory cells specific to tumor alloantigens. Screening tests in vitro showed that α-chain-centric clonotypes were contained in both repertoires, indicating that α-chain centricity could be an inherent feature of some TCRs. In both repertoires, the proportions of such clonotypes were found to be 20% to 25% of clonotypes expanded during antigenic stimulation. Transduction of naïve T cells with the individual dominant-active TCRα from both repertoires generated cytotoxic effectors that specifically killed the cognate tumor cells in vitro. This study confirmed previous findings that T cell modification with TCRα of α-chain-centric clonotypes generated functionally active antigen-specific effectors. Clonotypes with the dominant-active TCRα were actively involved in the primary allogeneic response. In contrast to primarily activated effectors, α-chain-centric memory clonotypes had the complementarity-determining region 3 (CDR3) with the unique physicochemical signature of shorter length, increased charge, and lower volume and polarity. Here, α-chain-centric memory clonotypes were proposed as promising candidates for adoptive TCR–T cell therapy.

## Graphical Abstract

**Figure:**
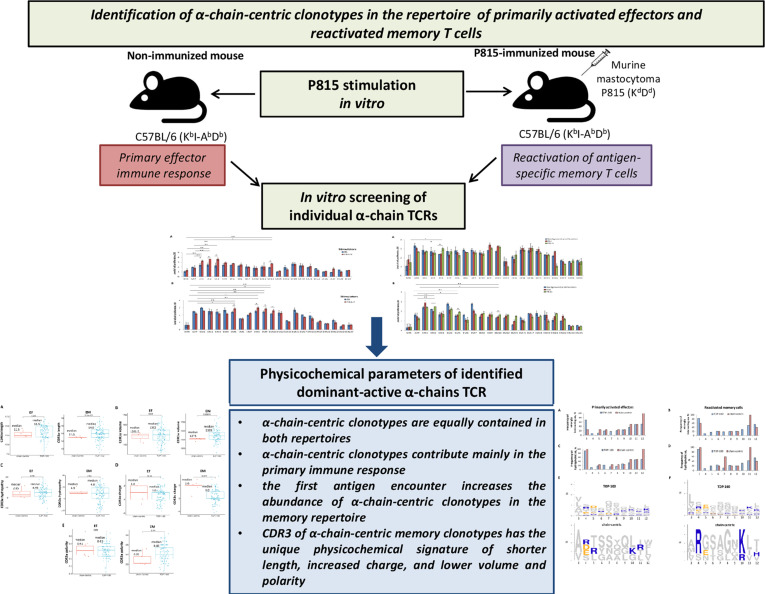

## Introduction

The phenomenon of T cell receptor (TCR) chain centricity has been acknowledged for decades [1]. This unique feature of some TCRs, i.e., the dominant role of one hemi-chain (a dominant-active α- or β-chain) in antigen recognition and peptide/major histocompatibility complex (pMHC) interactions, could be advantageous for T cell gene modification techniques to generate therapeutic T cell products for different clinical applications, including cancer therapy [1,2].

Some key issues regarding TCR chain centricity have yet to be discovered. Firstly, the exact proportion of chain-centric receptors in the native TCR repertoire is unknown. Previously, we showed that a naturally formed pool of memory T cells contained a substantial fraction of chain-centric TCRs (20%) [2], but it is not completely clear if other functional T cell subsets (i.e., antigen-inexperienced cells and effectors) could also express such receptors. This raises the question of the role of T cell antigen encounter in the selection or formation of chain-centric TCRs. Solving these fundamental problems will improve the identification and isolation of T cells with chain-centric TCRs, which could be beneficial for adoptive T cell therapy [1,2].

Although studies have proven that any hemi-chain TCR can dominate in antigen recognition [1], the ratio and frequency of α- and β-centric TCRs remain unknown. Still, α-chain-centric TCRs may prevail, taking into account some physiological and functional aspects of α-chain TCR (TCRα) [1]. This supposition could be supported by our recent bioinformatics study of TCR physicochemical features in mouse primarily activated effectors and reactivated memory cells specific to tumor antigens [3]. It was shown that, compared to primarily activated effectors, the repertoire of reactivated memory T cells was enriched with clonotypes whose TCRs contained α-chains with increased interaction strength and presumed higher cross-reactivity to multiple antigens [3]. Of particular note were minor changes in TCRβ physicochemical features in the repertoire of reactivated memory cells, implying the dominant role of TCRα in tumor antigen recognition during the secondary immune response [3].

**Fig. 1.**
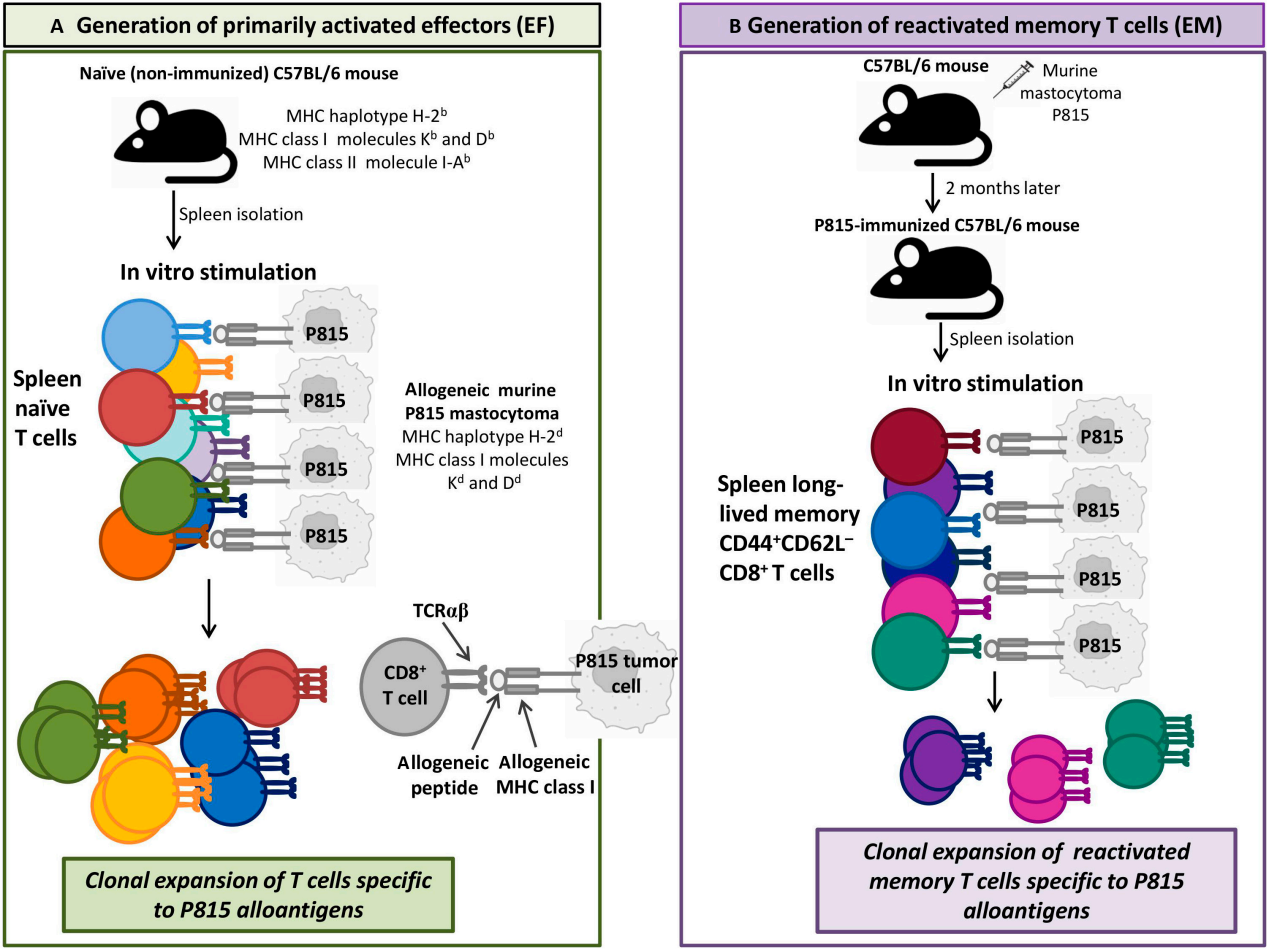
The experimental design. A schematic presentation of the workflow for the generation of primarily activated effectors (EF) (A) and reactivated memory T cells (EM) (B). (A) The spleen was recovered from naïve C57BL/6 mice (the MHC haplotype H-2^b^), and spleen cells were cultured in vitro with P815 mastocytoma cells (the MHC haplotype H-2^d^). T cells that recognized allogeneic MHC class I/peptide complexes expanded and generated a pool of primarily activated effectors. (B) Naïve C57BL/6 mice were immunized with P815 mastocytoma. Due to a mismatch in MHC class I molecules between recipient mice (K^b^D^b^) and tumor cells (K^d^D^d^), P815 was rejected in mice, and a pool of long-lived CD44^+^CD62L^−^ CD8^+^ memory T cells formed. Two months after in vivo immunization, the spleen was recovered from the P815-immunized mice, and memory T cells were rechallenged with the same P815 tumor. This resulted in the clonal expansion of reactivated memory T cells specific to tumor alloantigens.

Based on these primary findings, in this study, we aimed at evaluating the abundance of α-chain-centric clonotypes in the repertoire of primarily activated effectors and reactivated memory T cells. For this, we used an experimental model of induction of the immune response in C57BL/6 (B6) mice (the H2-K^b^ haplotype) to allogeneic mastocytoma P815 (K^d^D^d^), as described in our initial work [3]. After in vivo immunization with P815 cells, long-lived CD8^+^CD44^+^CD62L^−^ memory T cells generate in mice and can be reactivated in vitro by cognate antigenic stimulation [3]. T cells from naïve (without prior exposure to P815) B6 mice also respond to P815 cells, and this induces primarily activated CD8^+^ effectors [3,4]. Hence, we generated primarily activated effectors (EF) by in vitro stimulation of splenocytes from naïve (non-immunized) B6 mice with P815 tumor cells expressing allogeneic pMHC complexes, which resulted in expansion of antigen-specific clonotypes (Fig. 1A) [3]. Reactivated memory cells (EM) were generated by in vitro re-exposure of splenocytes from P815-immunized B6 mice to the same P815 tumor with expansion of memory clonotypes specific to the alloantigens (Fig. 1B) [3]. TCRα repertoires were then analyzed individually for EF and EM clonotypes [3]. Next, we selected unique TCRα variants from the repertoires of primarily activated effectors and reactivated memory cells by their increased frequencies compared to the respective repertoires of non-activated cells. To determine dominant-active antigen-specific α-chains among chosen TCRα variants (i.e., α-chain-centric TCR clonotypes), they were evaluated in several in vitro functional assays. Our findings here showed that α-chain-centric TCRs could be identified in the repertoires of both primarily activated effectors and memory T cells.

## Materials and Methods

### Animals

Female and male C57BL/6 (H-2^b^) (B6) and DBA/2 (H-2^d^) mice (18 to 22 g, 6 to 8 weeks of age) were obtained from the breeding facility of N.N. Blokhin National Medical Research Center of Oncology of the Ministry of Health of the Russian Federation (N.N. Blokhin NMRCO, Moscow, Russia). Mice were housed in facilities maintained at 20 to 24 °C with a 40% relative humidity and a 12-h light/dark cycle. All mice had ad libitum access to standard rodent chow and filtered tap water. Mice were handled in strict compliance with the NIH Guide for the Care and Use of Laboratory Animals (8th edition, 2011). All the experimental procedures were approved by the Ethics Committee on Animal Experimentation of N.N. Blokhin NMRCO.

### Tumor cell lines

Murine mastocytoma P815 (K^d^D^d^) [TIB-64, American Type Culture Collection (ATCC)], P815-GFP, lymphoma EL4 (K^b^D^b^) (TIB-39, ATCC), and EL4-GFP were obtained from the collection of N.N. Blokhin NMRCO and cultured in RPMI 1640 medium (PanEco, Moscow, Russia) supplemented with 10% fetal bovine serum (HyClone, GE Healthcare, Chicago, IL), 0.01 mg/ml ciprofloxacin (KRKA, Novo Mesto, Slovenia), and 0.01 M Hepes (PanEco) (complete RPMI). Tumor cells at 70% confluence were harvested, counted in a hemocytometer after trypan blue/eosin (mixed 1:1 v/v) staining, and used in mixed lymphocyte-tumor cultures (MLTCs) and the CTL-test as described below.

### Generation of TCRα cDNA libraries

The generation of TCRα cDNA libraries of primarily activated effectors and reactivated memory T cells was described in detail in our initial work [3]. Briefly, spleen cells recovered from non-immunized and P815-immunized B6 mice were stimulated in vitro with P815 cells to generate primarily effectors and reactivated memory T cells, respectively. After coculture, RNA was isolated from the spleen cells, and TCRα cDNA libraries of primarily activated effectors and reactivated memory cells were prepared as described elsewhere [5]. Next-generation sequencing was performed on the MiSeq platform (Illumina, San Diego, CA) using the Myseq reagent kit (300 cycles). Raw data were processed using MiGEC [6]. Further clonotype extraction from the MiGEC-assembled data was performed using MiXCR software [7]. Similarly, TCRα cDNA libraries of non-immunized and P815-immunized B6 mice without in vitro stimulation were generated. As a result, 2 cDNA libraries were generated for each non-immunized and P815-immunized mouse that contained TCRα clonotypes with or without in vitro stimulation by P815. Next, 2 cDNA libraries (with and without stimulation) of each non-immunized and P815-immunized mouse were compared, and clonotypes with the increased frequency after stimulation were selected for subsequent TCRα cloning (Table S1).

### TCRα cloning and retrovirus production

Twenty unique variants of TCRα of primarily activated effectors and reactivated memory cells were selected by their enriched frequency after P815 stimulation, as listed in Table S1. The cloning of individual TCRα was described previously in detail [2]. The full-length cDNA of TCRα or green fluorescent protein (GFP) was cloned into the MigRI retroviral vector under the phosphoglycerate kinase (PGK) promoter. All 40 cloned variants of TCRα were sequenced and compared with the predicted sequences to avoid mismatches (Supplementary Data 1). The pCL-Eco plasmid, kindly provided by Beliavskiĭ A.V. (Engelhardt Institute of Molecular Biology, Russian Academy of Sciences, Moscow, Russia), was used as a packaging plasmid for retroviruses. The 293T cell line was transfected with the plasmids by the calcium-phosphate method as described elsewhere [2,8].

### T cell transduction

T cell retroviral transduction was performed as previously described [2,8]. Briefly, the spleen and lymph nodes were isolated from non-immunized (intact) B6 mice, and cells were gently squeezed from the organ stroma in a Potter homogenizer in 3 ml of phosphate-buffered saline (PBS). Recovered cells were activated in vitro with 3 μg/ml concanavalin A (Sigma-Aldrich, St. Louis, MO) and 10 U/ml mouse interleukin-2 (IL-2) (Sigma-Aldrich) for 24 h. Activated T cells (both CD4^+^ and CD8^+^) were then transduced with the retrovirus containing the gene of the individual TCRα by 2 rounds of spinoculation at 2,000*g* for 90 min. The endogenous TCRαβ gene expression was not modified, facilitating random pairing of the transduced TCRα with various TCRβ. The efficiency of transduction was determined on days 2 to 3 post-transduction using flow cytometry by measuring GFP fluorescence in parallel control probes similarly transduced with the retrovirus containing the GFP gene [2,8]. The transduction efficiency was 60 ± 15% (data not shown). Nontransduced T cells (NTR), similarly activated and cultured as TCRα-modified T cells, were used as the background control in the in vitro functional assays.

### Mixed lymphocyte reaction

T cells transduced with individual TCRα or GFP (1 × 10^5^ cells per well) were seeded in triplicates in 96-well U-bottom plates (Corning Costar, Sigma-Aldrich) in 100 μl of complete RPMI 1640 supplemented as described above with the addition of 10 μM 2-mercaptoethanol (Merck, Darmstadt, Germany) (the supplemented complete medium). Spleen cells recovered from non-immunized B6 and DBA/2 mice were used as syngeneic and specific allogeneic stimulators, respectively. For this, stimulators were treated with mitomycin C (MitC) (Kyowa Hakko Kogyo Co. Ltd., Japan) (25 μg/ml, 37 °C, 45 min) and washed 3 times in PBS by centrifugation (200*g*, 5 min, 4 °C). Stimulators were then added to responder transduced T cells at 3 × 10^5^ cells per well in 100 μl of the supplemented complete medium to the final volume of 200 μl. Cells were cultured at 37 °C with 5% CO_2_ for 72 h. NTR cells were similarly seeded and cultured with MitC-treated stimulators. The level of cell proliferation was measured using CellTiter 96 AQueous Non-Radioactive Cell Proliferation Assay (Promega, Madison, WI) according to the manufacturer’s recommendations. The level of cell proliferative activity was expressed as optical density (OD). An α-chain TCR was considered dominant-active if the proliferation level of T cells modified with this TCRα in response to DBA/2 significantly exceeded both the background proliferation (in the presence of syngeneic B6 splenocytes) of these T cells and the proliferation level of control (NTR and GFP-transduced) T cells in response to DBA/2. Mixed lymphocyte reaction (MLR) was performed at least in 2 biological repeats.

### Mixed lymphocyte-tumor culture

TCRα- or GFP-modified T cells were seeded in triplicates in 96-well flat-bottom plates (Corning Costar, Sigma-Aldrich) as described above. EL4 and P815 cells were treated with MitC (50 μg/ml, 37 °C, 60 min) as described above and used as syngeneic and specific allogeneic stimulators, respectively. For this, 5 × 10^4^ stimulator cells per well were added to responder transduced T cells (at a ratio of 1:2) in 100 μl of the supplemented complete medium to the final volume of 200 μl. Cells were cultured at 37 °C with 5% CO_2_ for 72 h. NTR cells were similarly seeded and cultured with MitC-treated stimulator tumor cells. NTR cells and TCRα- or GFP-transduced T cells similarly cultured alone were used to evaluate the background cell proliferation. The level of cell proliferation was measured as described above. An α-chain TCR was considered dominant-active if the proliferation level of T cells modified with this TCRα in response to P815 significantly exceeded the background and EL4-induced proliferation of these T cells and the proliferation level of control (NTR and GFP-transduced) T cells in response to P815. MLTC was performed at least in 2 biological repeats.

### CTL-test

NTR cells and TCRα-modified T cells (6 × 10^5^ cells per well) were seeded in 24-well plates (Corning Costar, Sigma-Aldrich) in 1 ml of the supplemented complete medium. P815-GFP cells were added to responders at 3 × 10^5^ cells per well in 1 ml of the supplemented complete medium to the final volume of 2 ml. Similarly, NTR and TCRα-modified T cells were cultured with EL4-GFP cells as a control. Cells were cultured at 37 °C with 5% CO_2_ for 24 h. Cells were then harvested and stained with propidium iodide (PI) (75 μM; Sigma, St. Louis, MO) to detect dead cells. The level of cytotoxic activity was analyzed by flow cytometry using FACSCanto II (BD Bioscience, Franklin Lakes, NJ) with the FACSDiva 6.0 software (BD Bioscience) by evaluating the percentages of PI^+^ GFP^+^ tumor cells in cultures. The CTL-test was performed at least in 2 biological repeats.

### Bioinformatics analysis

The probability of complementarity-determining region 3 (CDR3) sequence generation (Pgen) of each selected TCRα clonotype was calculated using the tcrdist3 package as described elsewhere [9]. The fold enrichment of the selected TCRα clonotype of primarily activated effectors and reactivated memory cells after stimulation was calculated as a ratio of its frequency before and after antigenic stimulation (Table S1).

### Analysis of physicochemical properties of TCRα CDR

The physicochemical properties of amino acids in the CDR3 region were analyzed for each selected TCRα of primarily activated effectors (EF) and reactivated memory cells (EM) (Table S1). Evaluations were performed using VDJtools [10]. Hydropathy, strength (an estimated indirect bioinformatics value of interaction affinities between amino acid pairs at the CDR3–peptide interface), charge, volume, and polarity were calculated for the full CDR3 region of each selected EF and EM TCRα clonotype. These values were then compared with the corresponding characteristics of the TOP-100 most frequent clonotypes (as defined by the frequency) in the relevant repertoires. Additionally, the strength of the 5 central amino acids of the CDR3 loop (cCDR3) and the cumulative strength of CDR1 and CDR2 were calculated for each identified dominant-active α-chain TCR.

### Statistical analysis

Data are presented as mean ± standard deviation (SD) or mean ± standard error of the mean (SEM). All statistical analyses were performed using the unpaired Student’s *t* test and one-way analysis of variance (ANOVA). A *P* value of <0.05 was considered significant. All statistical analyses were performed using the Prism software (v. 8.1.2, GraphPad, San Diego, CA) and SRplot [11].

## Results

### Selection of TCRα clonotypes from the repertoire of primarily activated effectors and reactivated memory cells for cloning and functional testing

To identify α-chain-centric TCR clonotypes in the repertoire of primarily activated effectors (EF) and reactivated memory T cells (EM), 20 α-chains were selected from each repertoire based on their enrichment after antigenic stimulation (Table S1) [3]. The fold enrichment was calculated as a ratio of the clonotype frequency after antigenic stimulation to its frequency before stimulation and indicated the relative involvement of the respective clonotype in the immune response (the primary immune response for EF clonotypes and the secondary immune response for EM clonotypes). The physicochemical characteristics of the selected EF and EM TCRα clonotypes did not markedly differ from the physicochemical characteristics of the TOP-100 clonotypes in the respective repertoire (Fig. S1), showing unbiased selection of TCRα clonotypes for cloning and functional testing.

### α-Chain-centric TCRs contained in the repertoire of primarily activated effectors and reactivated memory cells

**Fig. 2.**
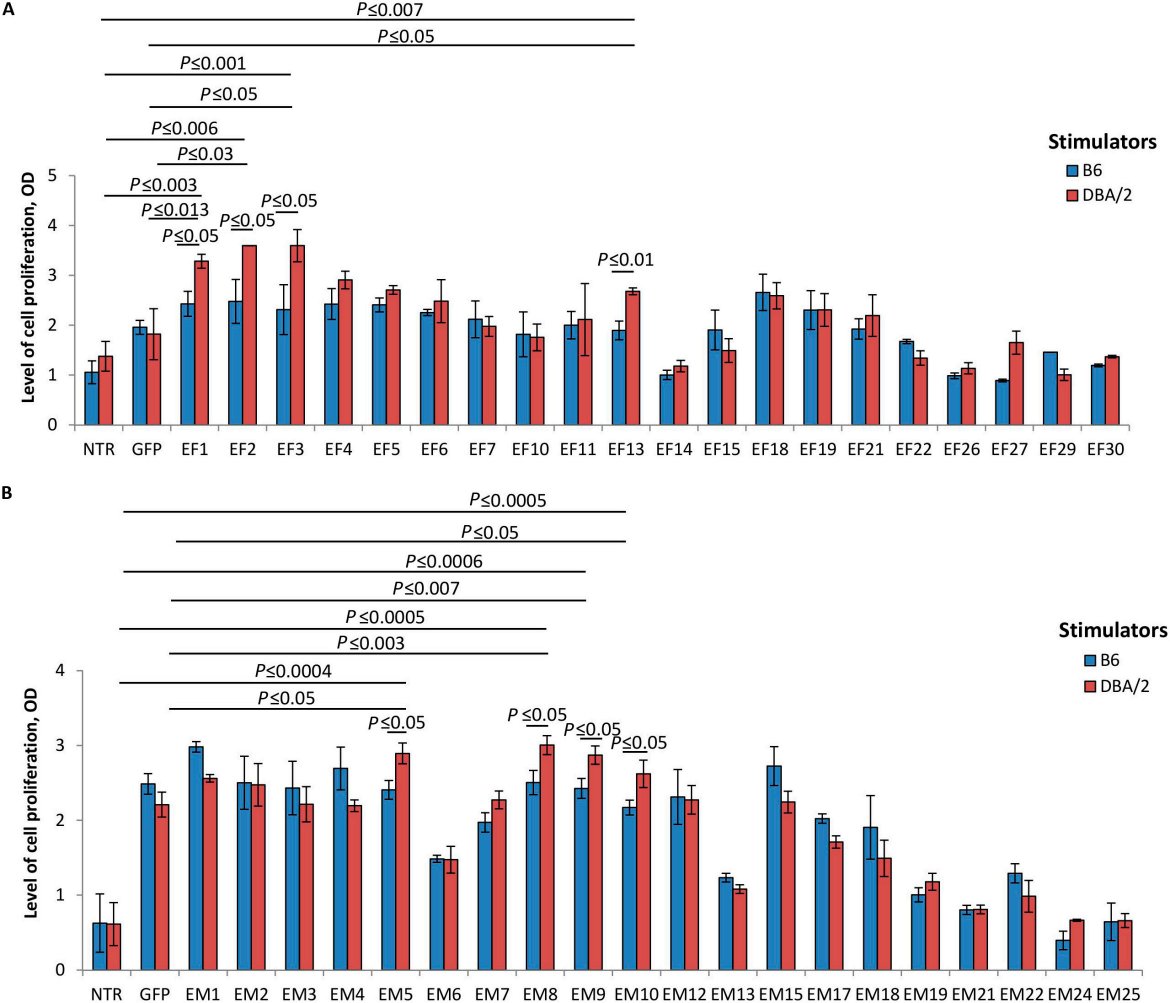
In vitro screening of individual α-chain TCRs from the repertoires of primarily activated effectors and reactivated memory cells in MLR. Twenty α-chain TCRs (TCRα) were selected from the repertoire of primarily activated effectors (EF) or reactivated memory T cells (EM), generated as described in Materials and Methods. T cells recovered from intact C57BL/6 (B6) mice were transduced with the individual variant of the selected TCRα and cultured with mitomycin C (MitC)-treated cognate allogeneic (DBA/2) splenocytes. TCRα-modified T cells were similarly cultured with MitC-treated syngeneic (B6) splenocytes to evaluate the background proliferation. Nontransduced (NTR) T cells and GFP-modified T cells, similarly cultured in MLR, were used as negative controls. The proliferative response (OD) in MLR of T cells transduced with TCRα from the EF repertoire (A) or the EM repertoire (B) was evaluated after 72 h. The data of one of 2 representative experiments was shown as mean ± SD for 3 technical repeats. One-way ANOVA corrected with Tukey’s multiple comparison.

**Fig. 3.**
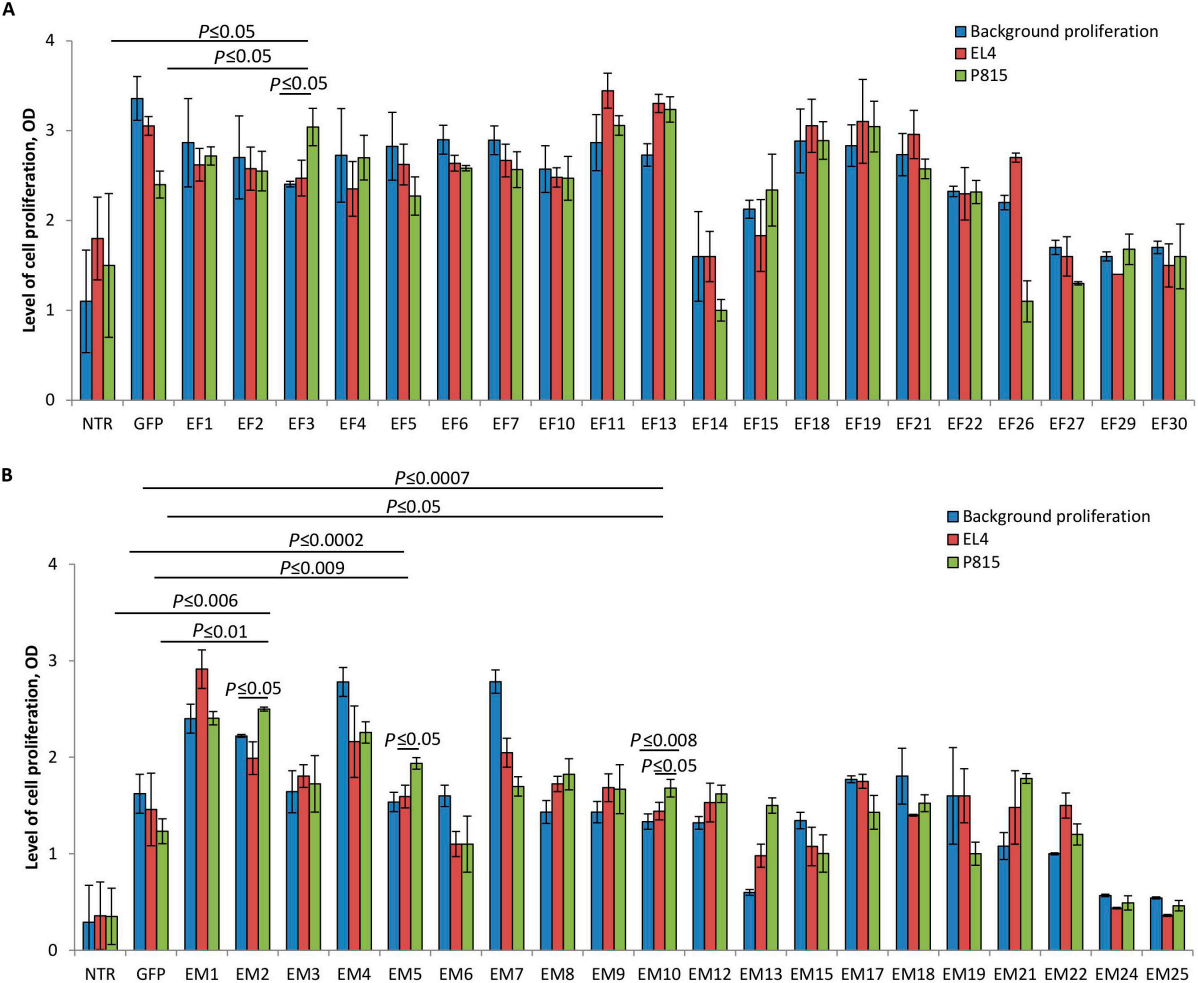
T cells transduced with the individual α-chain TCR from the repertoires of primarily activated effectors and reactivated memory cells responded to P815 alloantigens in vitro. Twenty α-chain TCRs (TCRα) selected from the repertoire of primarily activated effectors (EF) or reactivated memory T cells (EM) were transduced into T cells from intact C57BL/6 mice. TCRα-modified T cells were then cultured with mitomycin C (MitC)-treated cognate allogeneic mastocytoma P815. Transduced T cells were similarly cultured alone or with MitC-treated syngeneic lymphoma EL4 to evaluate the background proliferation. Nontransduced (NTR) T cells and GFP-modified T cells, similarly cultured in MLTC, were used as negative controls. The proliferative response (OD) in MLTC of T cells transduced with TCRα from the EF repertoire (A) or the EM repertoire (B) was evaluated after 72 h. The data of one of 2 representative experiments were shown as mean ± SD for 3 technical repeats. One-way ANOVA corrected with Tukey’s multiple comparison.

Genes of the selected TCRα (Table S1) were individually transduced into activated T cells of intact (non-immunized) B6 mice (H-2^b^) with the unmodified endogenous TCRαβ repertoire, ensuring random pairing of the transferred α-chain with different endogenous β-chains. TCRα-modified T cells were then tested in vitro for their ability to recognize cognate alloantigens in MLR and MLTC with DBA/2 spleen cells (H-2^d^) and mastocytoma P815 (K^d^D^d^) used as the specific stimulators, respectively (Figs. 2 and 3).

In MLR, 4 variants of T cells modified with TCRα from the EF repertoire (EF1, EF2, EF3, and EF13) showed the enhanced proliferative response to the specific DBA/2 stimulators, which significantly exceeded their background proliferation (in response to B6) and DBA/2-induced proliferation of control NTR and GFP-transduced T cells (Fig. 2A). These data indicated that EF1, EF2, EF3, and EF13 were dominant-active antigen-specific α-chain TCRs and dictated the response to the cognate alloantigens when transduced into naïve (P815-unprimed) T cells. Similarly, the EM repertoire contained 4 dominant-active TCRα (EM5, EM8, EM9, and EM10), as revealed in MLR (Fig. 2B). T cells transduced with these individual α-chains responded to DBA/2 significantly stronger versus the respective controls (Fig. 2B).

Next, TCRα-transduced T cells were screened in MLTC with P815 cells (Fig. 3). This functional assay showed that T cells transduced with EF3 responded to the cognate P815 stimulation: the proliferation level of EF3-transduced T cells in cultures with P815 was significantly higher compared to the background and EL4-induced proliferation of these cells as well as the proliferative responses of NTR and GFP-modified T cells to P815 (Fig. 3A). T cells modified with 3 individual TCRα from the EM repertoire (EM2, EM5, and EM10) specifically responded to P815 alloantigens in MLTC (Fig. 3B).

These in vitro tests showed the functional dominance of some α-chains originated from the TCR repertoire of primarily activated effectors and reactivated memory T cells in a polyclonal β-chain context, indicating that both EF and EM repertoires contained clonotypes with α-chain-centric TCRs. The proportion of such clonotypes was comparable in these 2 repertoires: 4 of 20 selected enriched clonotypes (20%) in the EF repertoire (Figs. 2A and 3A) and 5 of 20 selected enriched clonotypes (25%) in the EM repertoire (Figs. 2B and 3B).

### The relative contribution of α-chain-centric TCR clonotypes to the primary and secondary immune responses

**Fig. 4.**
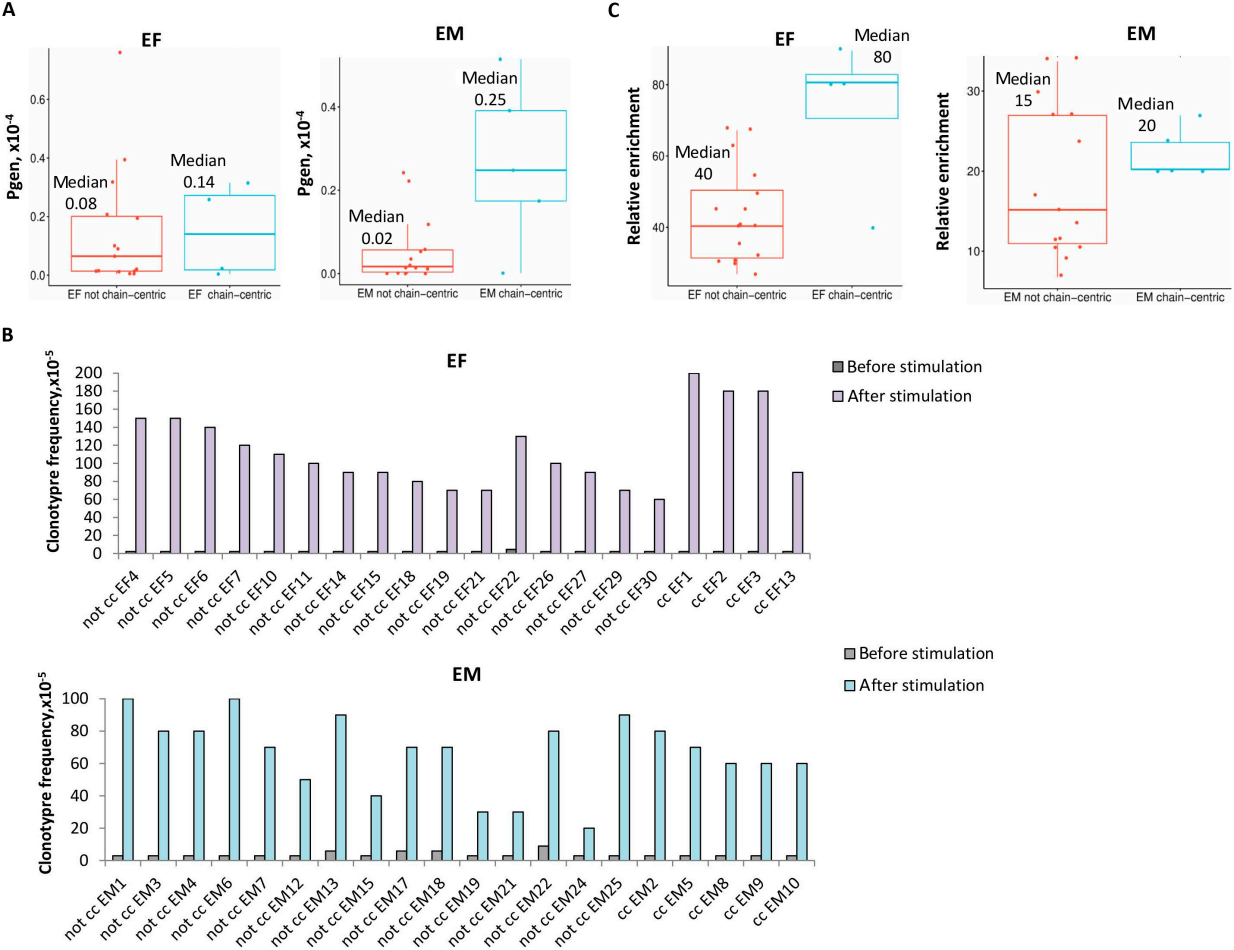
The relative involvement of α-chain-centric clonotypes in the primary and secondary immune responses. Clonotypes with α-chain-centric TCRs were identified in the repertoire of primarily activated effectors (EF) and reactivated memory T cells (EM) in functional tests in vitro (Figs. 2 and 3). (A) The probability of CDR3 sequence generation (Pgen, ×10^−4^) was calculated for α-chain-centric EF and EM clonotypes and compared with Pgen of non-chain-centric clonotypes selected in the respective repertoire for functional in vitro testing (Table S1). (B) The frequency (×10^−5^) of non-chain-centric (not cc) and chain-centric (cc) EF and EM clonotypes before and after antigenic stimulation. (C) The relative enrichment of non-chain-centric and α-chain-centric EF and EM clonotypes was calculated as a ratio of the clonotype frequency after antigenic stimulation to its frequency before stimulation. The median relative enrichment of α-chain-centric EF and EM clonotypes was compared to those of non-chain-centric clonotypes in the respective repertoire.

To evaluate the involvement of α-chain-centric EF and EM clonotypes in the primary and secondary immune responses, respectively, their relative enrichment was calculated and compared to the respective values of non-chain-centric TCRα clonotypes among the selected 20 variants (Fig. 4 and Table S1). Additionally, Pgen was calculated individually for non-chain-centric and α-chain-centric EF and EM clonotypes, which reflects the probability of CDR3 stochastic generation and its presumed belonging to the functional repertoire.

We found that the median Pgen of α-chain-centric EF clonotypes tended to increase compared to the median Pgen of non-chain-centric EF clonotypes (Fig. 4A). Interestingly, α-chain-centric memory clonotypes (EM) had the highest median Pgen compared to the Pgen of both non-chain-centric EM clonotypes and α-chain-centric EF clonotypes (Fig. 4A).

To analyze the relative contribution of α-chain-centric clonotypes to the immune response, their fold enrichment was calculated as a ratio of the clonotype frequency after antigenic stimulation to its frequency before stimulation (Fig. 4B and C and Table S1). The primary antigenic stimulation induced robust expansion of non-chain-centric EF clonotypes with a median 40-fold enrichment. Interestingly, α-chain-centric EF clonotypes were 80-fold enriched after primary stimulation (Fig. 4C). The fold enrichments of α-chain-centric and non-chain-centric EM clonotypes were comparable and markedly lower versus EF clonotypes (Fig. 4C). Notably, both Pgen and fold enrichment of non-chain-centric EM clonotypes were lower compared to the respective values of non-chain-centric EF clonotypes (Fig. 4). These data correlated well with our initial bioinformatics analysis of TCR repertoires that showed that more unique antigen-specific clonotypes with lower Pgen selectively expanded in response to the specific antigenic restimulation [3].

These findings suggested that α-chain-centric clonotypes were actively involved in the primary response. In contrast, based on fold enrichment (Fig. 4B and C), α-chain-centric clonotypes contributed to the secondary response equally to non-chain-centric clonotypes.

### T cells modified with the dominant-active TCRα from EF and EM repertoires displayed cytotoxic activity against P815

**Fig. 5.**
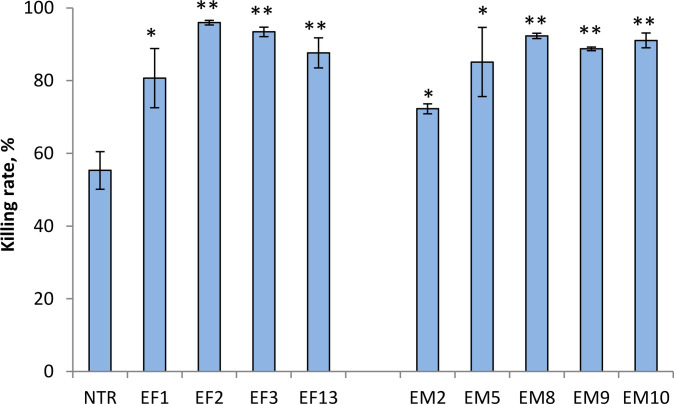
T cells transduced with the dominant-active α-chain TCRs killed cognate tumor cells in vitro. T cells of intact C57BL/6 mice (H-2^b^) were modified with identified dominant-active TCRα from the repertoire of primarily activated effectors (EF) or reactivated memory T cells (EM) (Figs. 2 and 3). Transduced T cells were mixed with the allogeneic mastocytoma P815-GFP (K^d^D^d^) at a ratio of 2:1. Nontransduced (NTR) T cells were used as the control. Cytotoxic activity of transduced T cells was analyzed by flow cytometry after 24 h of culturing by evaluating the percentage of dead P815 tumor cells as GFP^+^PI^+^ cells. Data from 2 to 4 independent experiments are shown as mean ± SD. *P < 0.05, **P < 0.01 versus the NTR control, unpaired Student’s *t* test.

To further evaluate the functional activity of dominant-active α-chains, T cells from intact (P815-unprimed) B6 mice were transduced with the identified dominant-active α-chain TCRs from the EF or EM repertoires (EF1, EF2, EF3, EF13 and EM2, EM5, EM8, EM9, EM10, respectively) (Figs. 2 and 3). The cytotoxic activity of modified T cells against P815 cells was then analyzed in the CTL-test in vitro (Fig. 5). All TCRα-modified T cells were able to kill P815, and their killing rates were significantly higher versus control (NTR) T cells (Fig. 5). Notably, no cytotoxicity of TCRα-transduced T cells was detected in the cultures with syngeneic EL4 cells (Fig. S2), indicating that P815 killing was specific. Thus, modification of T cells with the dominant-active antigen-specific TCRα generates cytotoxic T cells that specifically kill cognate targets.

### Interaction strength of CDRα could influence allorecognition by α-chain-centric TCR

Of particular note is our finding that T cells modified with dominant-active TCRα from either the EF or EM repertoire differently responded in MLR and MLTC to the cognate stimulators (Table S2). T cells individually transduced with 4 dominant-active TCRα from the repertoire of primarily activated effectors (EF1, EF2, EF3, EF13) responded in MLR to DBA/2 alloantigens (Fig. 2A), but only EF3-modified T cells could also respond to P815 in MLTC (Fig. 3A). Similarly, EM5- and EM10-transduced T cells responded to both DBA/2 and P815 (Figs. 2B and 3B), while EM2-transduced T cells responded only in MLTC, and EM8- and EM9-modified T cells proliferated in MLR but not in MLTC (Figs. 2B and 3B and Table S2).

**Fig. 6.**
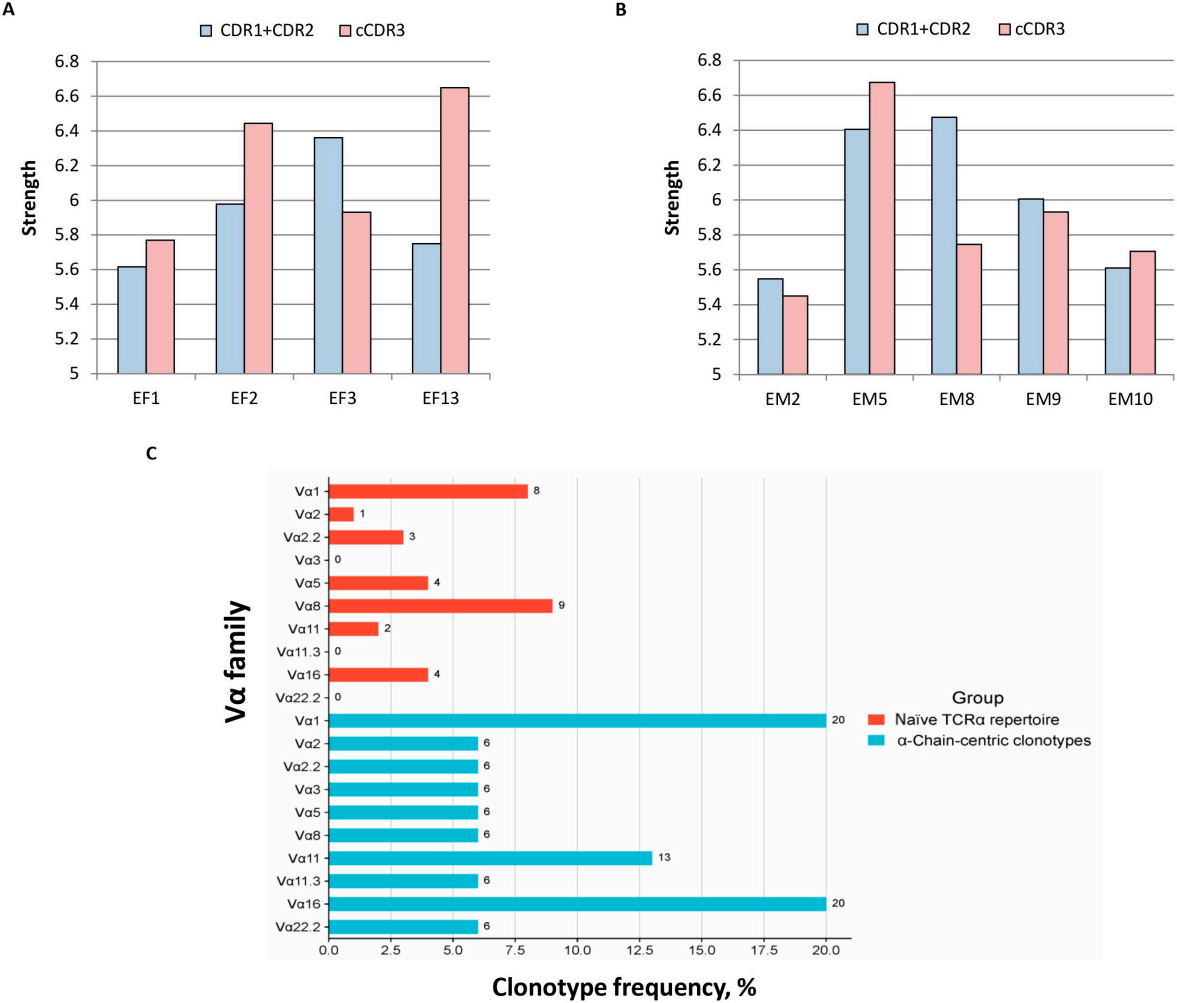
Interaction strength of the CDR loops of dominant-active α-chain TCRs. Dominant-active α-chain TCRs in the repertoire of primarily activated effectors (EF) and reactivated memory cells (EM) were identified in MLR and MLTC (Figs. 2 and 3). The cumulative interaction strength of CDR1 and CDR2 and the interaction strength of the 5 central amino acids of CDR3 (cCDR3) were calculated for each dominant-active EF (A) and EM (B) α-chain TCR. (C) The comparative analysis of the use of Vα families (%) by α-chain-centric clonotypes, identified in this study and previous research [2,17], and the TOP-100 largest clonotypes in the naïve B6 TCRα repertoire [3].

It is acknowledged that allorecognition depends mainly on direct interactions of TCR with an allogeneic MHC molecule and a docked peptide [12–14], which are mediated by CDR1 + CDR2 and CDR3 of TCR, respectively [15,16]. Therefore, the observed discrepancy in MLR and MLTC responses of TCRα-modified T cells could be attributed to physicochemical parameters of CDR regions of identified dominant-active α-chains that could dictate the mode of allo-MHC-peptide recognition. To ascertain this, for each identified dominant-active α-chain, we calculated the interaction strength of its CDR1 + CDR2 and of 5 central amino acids of CDR3 (cCDR3) that predominantly interacts with a docked peptide [16] (Fig. 6).

EF2 and EF13 α-chains had higher cCDR3 strength compared to the respective CDR1 + CDR2 strength. In contrast, α-chain EF3 possessed higher CDR1 + CDR2 strength versus its cCDR3 (Fig. 6A). This could indicate that α-chains EF2 and EF13 interacted more strongly with a docked peptide, while EF3, having a higher cumulative strength of CDR1 + CDR2, could interact more strongly with allo-MHC molecules. Therefore, in transduced T cells, EF2 and EF13 α-chains could generate allorestricted TCRs that specifically recognize peptide(s) presented by allo-MHC molecules (H-2^d^). Since the repertoire of peptides presented by both MHC class I and II molecules on DBA/2 spleen cells could hypothetically be broader than the P815 peptide repertoire presented only by MHC class I molecules (H-2K^d^ and H-2D^d^), this could explain why EF2- and EF13-transduced T cells proliferated only in MLR (in response to DBA/2 splenocytes) (Fig. 2A) but not in MLTC (in response to P815) (Fig. 3A). In contrast, EF3 α-chain could generate alloreactive TCRs in transduced T cells that recognized alloantigens in the peptide-independent manner, and, hence, EF3-modified T cells proliferated in MLR and MLTC (Figs. 2A and 3A), responding to the same allo-MHC class I molecules expressed on both nonmalignant DBA/2 splenocytes and P815 tumor cells.

In contrast to TCRα from the repertoire of primarily activated effectors (Fig. 6A), the CDR1 + CDR2 and cCDR3 strengths were nearly equal in all but one (EM8) α-chains from the EM repertoire (Fig. 6B). These data finely correlated with our initial findings of the increased interaction strength of the germline Vα segments and CDR3α of reactivated memory T cells [3], implying that during the secondary response, TCRα of memory cells strongly interacts with both the MHC molecule and a docked peptide. Thus, the CDRα strength alone could not account for the differential responses of EM-transduced T cells in MLR and MLTC (Figs. 2B and 3B), and other physicochemical characteristics of dominant-active TCRα from the memory repertoire could shape alloresponse in our functional tests.

### α-Chain-centric clonotypes belong to different Vα families

Identified α-chain-centric EF and EM clonotypes utilized TRAV gene segments (Table S1) that belong to different Vα families (Table S3). To analyze potential clustering of α-chain-centric clonotypes, we calculated their frequencies within each specified Vα family and compared them to the frequencies of the TOP-100 largest clonotypes belonging to the same Vα family in the naïve B6 TCRα repertoire [3] (Fig. 6C). Importantly, α-chain-centric clonotypes found in our previous studies using the EL4 tumor model [17] and the *Salmonella typhimurium* infection model [2] (Table S3) were incorporated into this comparison to enhance its robustness.

In total, 15 identified α-chain-centric clonotypes belonged to 10 Vα families (Table S3), with the highest clonotype frequencies detected in Vα1 (3 of 15, 20%), Vα11 (2 of 15, 13%), and Vα16 (3 of 15, 20%) families (Fig. 6C). Interestingly, of the TOP-100 TCRα clonotypes in the naïve B6 repertoire, only 8%, 2%, and 4% of clonotypes belonged to Vα1, Vα11, and Vα16 families, respectively (Fig. 6C). These data could suggest the relative enrichment of certain Vα families with α-chain-centric clonotypes. Subsequent research with larger datasets may validate this hypothesis and shed more light on the origin of such clonotypes.

### CDR3 of dominant-active TCRα in the EF and EM repertoires had distinct physicochemical characteristics

**Fig. 7.**
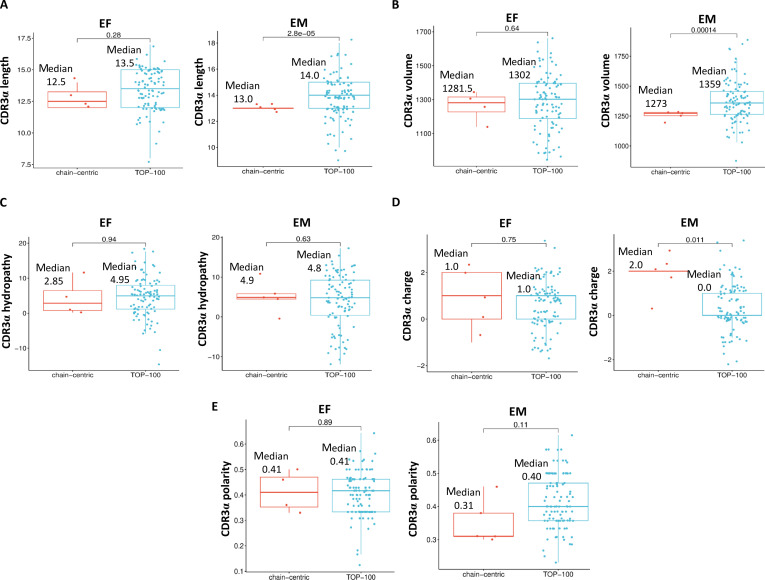
CDR3 physicochemical parameters of dominant-active α-chain TCRs. The length (A), volume (B), hydropathy (C), charge (D), and polarity (E) were calculated for dominant-active α-chain TCRs (chain-centric) in the repertoire of primarily activated effectors (EF) and reactivated memory cells (EM) and compared with the values of the TOP-100 TCRα clonotypes in the respective repertoire. Unpaired Student’s *t* test.

Next, physicochemical parameters (length, volume, charge, polarity, and hydropathy) of the CDR3 loop of the identified dominant-active TCRα from the EF and EM repertoires were analyzed and compared to those of the TOP-100 TCRα clonotypes in the respective repertoire (Fig. 7).

CDR3 of α-chain-centric EF clonotypes tended to be shorter (Fig. 7A) and contain a lower number of bulky amino acids (based on the median volume) (Fig. 7B) compared to the TOP-100 clonotypes in the respective repertoire. In α-chain-centric EM clonotypes, the CDR3 length and volume were significantly lower versus the respective values of the TOP-100 clonotypes in the repertoire of memory cells (Fig. 7A and B). The median CDR3 hydropathy of α-chain-centric EF clonotypes was 1.73-fold lower versus the value of the TOP-100 clonotypes in this repertoire (Fig. 7C). This parameter was not markedly changed in α-chain-centric EM clonotypes compared to the respective TOP-100 clonotypes (Fig. 7C). In contrast to EF clonotypes, CDR3 of α-chain-centric EM clonotypes had a significantly higher charge (Fig. 7D) and lower median polarity (Fig. 7E) versus the respective TOP-100 clonotypes.

### Features of amino acid distribution in CDR3 of dominant-active TCRα in the EF and EM repertoires

**Fig. 8.**
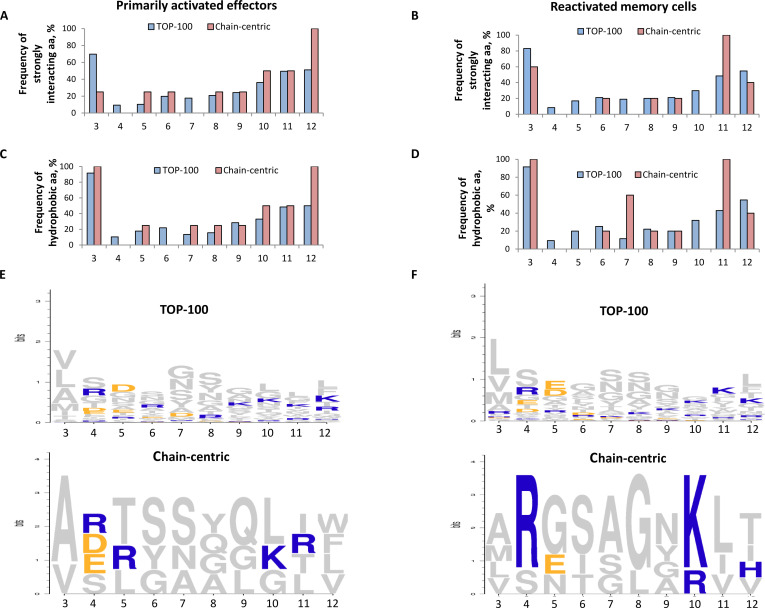
Features of amino acid utilization in the CDR3 loop of chain-centric TCRα clonotypes. The frequency (%) of strongly interacting amino acids (aa) (A and B) and hydrophobic aa (C and D) was calculated in positions 3 to 12 of the CDR3 loop of the identified dominant-active α-chain TCRs in the repertoire of primarily activated effectors (EF) (A and C) and reactivated memory cells (EM) (B and D). Logo plots for charged aa (gray, uncharged; yellow, negatively charged; blue, positively charged) in positions 3 to 12 of the CDR3 were created for α-chain-centric EF clonotypes (E) and α-chain-centric EM clonotypes (F). The respective parameters of the CDR3α region of the TOP-100 clonotypes in the EF and EM repertoires were used for the comparison.

The frequencies of strongly interacting (Fig. 8A and B) and hydrophobic (Fig. 8C and D) amino acids were calculated in each position of CDR3 of α-chain-centric EF and EM clonotypes and compared to the values of the respective TOP-100 clonotypes.

In the TOP-100 EF clonotypes, the CDR3 positions 3, 11, and 12 were enriched with strongly interacting and hydrophobic amino acids (Fig. 8A and C). In contrast, such amino acids were evenly distributed in the CDR3 positions 5 to 11 of α-chain-centric EF clonotypes (Fig. 8A and C), while the positions 3 and 12 were comprised entirely of strongly interacting and hydrophobic amino acids (W, F, L, V, A) (Fig. 8A, C, and E). Similarly, the CDR3 positions 3 and 11 of α-chain-centric EM clonotypes contained exclusively strongly interacting and hydrophobic amino acids (Fig. 8B and D). In addition, the CDR3 position 7 of α-chain-centric EM clonotypes was enriched with hydrophobic amino acids 5-fold versus the respective position in CDR3α of the TOP-100 EM clonotypes (60% versus 12%; Fig. 8D). Importantly, the fixed usage of positively charged amino acids R and K was found in the CDR3 positions 4 and 10 of α-chain-centric EM clonotypes (Fig. 8F). In position 4, R was utilized in 80% of chain-centric EM clonotypes compared to 25% of chain-centric EF clonotypes and only 1% of TOP-100 EM clonotypes. In position 10, the usage of positively charged R and K was detected in all chain-centric EM clonotypes versus 25% of chain-centric EF clonotypes and nearly 15% of TOP-100 EM clonotypes (Fig. 8E and F) (Supplementary Data 2). The preferential usage of R and K in α-chain-centric EM clonotypes could be linked to the increased CDR3 charge in these TCRα clonotypes (Fig. 7D).

## Discussion

This study indicated that α-chain-centric TCRs could be found in the repertoire of both primarily activated effectors and reactivated memory T cells. The frequency of such receptors was similar in both repertoires, accounting for 20% to 25% of the clonotypes that expanded in response to antigenic stimulation. This estimation correlated well with our previous findings [2]. Thus, α-chain centricity could be an inherent feature of some TCRs and generated during intrathymic T cell development either stochastically or as a result of TCR editing [1].

In the repertoire of primarily activated effectors, the probability of α-chain-centric clonotype generation (Pgen) tended to be higher than of non-chain-centric clonotypes, although without significant differences. Furthermore, the primary antigenic stimulation induced their intense enrichment. Interestingly, Pgen of α-chain-centric memory clonotypes was the highest among all analyzed clonotypes in both EF and EM repertoires, although they were equally involved in the secondary response as non-chain-centric memory clonotypes (Fig. 4). These findings suggested that α-chain-centric TCR clonotypes could intensively expand during the primary immune response and predominantly sustain during the contraction phase and the memory formation. As such, antigenic encounter could be a selective factor, enriching the memory repertoire with α-chain-centric clonotypes.

The identified dominant-active α-chain TCRs exhibited certain common physicochemical characteristics, such as a shorter CDR3 loop and reduced volume; however, the dominant-active TCRα of memory cells possessed the CDR3 physicochemical signature, distinct from that of the dominant-active α-chains of primarily activated effectors (Fig. 7). These results agreed with our recent TCR bioinformatics analysis, which showed that, unlike TCRβ, the CDRα physicochemical properties of reactivated memory cells changed markedly compared to those of primarily activated effectors [3]. Importantly, analysis of amino acid distribution in the CDR3 loop of α-chain-centric clonotypes suggested that in contrast to EF, CDR3 of α-chain-centric EM clonotypes could have a higher number of strong contacts with a docked peptide (at positions 3, 7, and 11 enriched with strongly interacting and hydrophobic amino acids), and these interactions could be stabilized by positively charged amino acids [18] at positions 4 and 10 (Fig. 8B, D, and F). Other studies also showed that CDR3α of α-chain-centric TCR had more contacts with the bound peptide [19]. Notably, in α-chain-centric EM clonotypes, the redistribution of strongly interacting and positively charged amino acids affected predominantly the CDR3α positions distal from the sites often interacting with the docked peptide (central 5 amino acids of CDR3 [16]). An α-chain-centric TCR with such CDR3α may require fewer important contacts to recognize the ligand because it may be less dependent on amino acid variations in the distal parts of the peptide presented in the MHC groove. Other studies suggested that this feature could indicate higher TCR cross-reactivity [20,21]. Still, the predicted cross-reactivity of identified α-chain-centric memory clonotypes must be directly verified in further studies.

Here, we propose that α-chain-centric memory clonotypes could be identified in a bulk repertoire based on the unique physicochemical signature of their CDR3 that includes a shorter length and lower volume and polarity, along with the increased charge. Additional studies in different experimental settings, in both mice and humans, could support the findings of this work and prove their generalizability.

In combination with other bioinformatics strategies for the identification of antigen-specific clonotypes, e.g., as previously described [22,23], the outcomes of this study could assist in developing algorithms for the identification and preselection of potentially therapeutic TCRα variants. Subsequently, such preselected clonotypes must be carefully verified in vitro to confirm their antigen specificity and check off-target reactivity. Our prior studies suggest that specifically α-chain-centric memory clonotypes may be a better source of therapeutic TCRs for adoptive cell therapy [2,3,17]. In experimental models, adoptive transfer of T cells transduced with the dominant-active antigen-specific α-chain TCR originated from memory cells provided in vivo therapeutic effects in infected or tumor-bearing hosts [3,17]. Furthermore, evaluations in preclinical models assured the safety of the TCRα-modified T cell product [24].

We hypothesize here that the physicochemical characteristics of an α-chain can supposedly also predict the mode of antigen recognition by α-chain-centric TCR. TCR recognizes allo-MHC-peptide complexes predominantly directly [12–14,25,26], i.e., allorecognition depends on direct TCR interactions with the allogeneic MHC molecule and a docked peptide. It is acknowledged that some alloreactive TCRs could interact with allo-MHC molecules irrespective of the presented peptide. In contrast, some TCRs are allorestricted, i.e., they specifically recognize a peptide presented by the allo-MHC molecule [27]. As such, in the case of alloreactive (peptide-independent) TCRs, one could expect higher interaction strength of their CDR1 and CDR2 that mediate interaction with MHC molecules [15] versus CDR3. As for allorestricted TCRs, the central 5 amino acids of their CDR3 that interact with a presented peptide [16] could supposedly have a higher interaction strength compared to their CDR1 and CDR2. The analysis of the CDR strength of identified dominant-active α-chain TCRs from the repertoire of primary activated effectors (Fig. 6A) partially corroborated this idea.

T cells modified with EF2 or EF13 TCRα with stronger interacting cCDR3 (Fig. 6A) responded only in MLR to DBA/2 alloantigens (Figs. 2A and 3A). Presumably, these α-chains could originate from allorestricted α-chain-centric TCRs and recognize the specific peptide(s) presented by allo-MHC molecules. In contrast, T cells transduced with the EF3 α-chain responded in both MLR and MLTC (Figs. 2A and 3A). Considering that this α-chain had the stronger interaction strength of CDR1 + CDR2 versus its cCDR3 (Fig. 6A), it could originate from the alloreactive α-chain-centric TCR and recognize allo-MHC class I molecules per se, independently of docked peptides. Future studies with structural modeling are required to corroborate the hypothesis made here on the predicted peptide-dependent and peptide-independent recognition by α-chain-centric TCRs.

Alternatively, we propose that the differential response of EF-modified T cells in functional in vitro assays (Figs. 2 and 3) could be due to the affinity of their TCRs generated after pairing of the transduced dominant-active α-chain with endogenous β-chains. In response to P815, T cells could not acquire sufficient costimulation since tumor cells do not provide any danger signals. Hence, the T cell proliferation in response to allogeneic tumor cells relied mainly on the strength of TCR interaction with pMHC that must be sufficient enough to achieve the activation threshold. In contrast, in response to allogeneic spleen cells in MLR, T cells received costimulatory signals from antigen-presenting cells in addition to TCR-mediated activation signals. All EF-modified T cells responded in MLR, but only one dominant-active EF α-chain (1 of 4; 25%) made recipient T cells proliferate in response to tumor alloantigens (EF3; Fig. 3A).

In contrast, T cells modified with 3 of 5 (60%) dominant-active α-chains from the memory repertoire proliferated in response to P815 tumor cells in MLTC (EM2, EM5, and EM10; Fig. 3B). It is known that memory cells are relatively independent of costimulation [28], have a lower activation threshold compared to naive T lymphocytes [29,30], and can respond to lower doses of the antigen [30–32]. These functional properties of memory cells could presumably be attributed to the features of their TCR. Indeed, it was shown that TCRs (and α-chains particularly) of memory T cells have a higher affinity for pMHC than TCRs of effectors involved in the primary response [3,33,34]. Thus, gene modification of T lymphocytes with α-chain-centric TCRs originated from memory cells could render functional features of true memory cells to recipient T cells, enabling them to directly recognize tumor alloantigens and expand (Fig. 3B). This was exquisitely confirmed in our TCRα-transgenic mouse model [17].

Taken together, studies here implied that the dominant-active α-chain could dictate the mode of antigen recognition by TCRα-modified T cells, further confirming the instructive role of the TCR structure in the development of T cell phenotype and functioning [17,35–37].

Importantly, T cells transduced with either EF or EM dominant-active α-chain TCR rapidly killed tumor cells expressing the cognate alloantigens (Fig. 5). This did not contradict the results of the MLTC test (Fig. 3), as T cell cytotoxic activity was shown not to be closely dependent on antigen-induced proliferation, and full activation was not required for T cells to accomplish their cytotoxic functions [38–41]. This study indicated that gene modification of naïve T cells with a single dominant-active TCRα of antigen-specific effectors or memory cells generated effectors ready to recognize and eliminate the specific targets. These outcomes fully complied with our previous research [2,17], further supporting the concept that α-chain-centric TCRs may represent promising candidates for T cell gene modification in adoptive cell therapy for cancer and infectious diseases [1–3,17,24].

## Data Availability

All data required to evaluate the conclusions of the study are provided in th e paper and the Supplementary Materials. The raw data will be made available upon reasonable request.
