# Supplementary material for: The Abundance of α-Chain-Centric TCRs in the Mouse Repertoire of Primarily Activated Effectors and Reactivated Memory T Cells
Source: Comput Struct Biotechnol J. 2026 Apr 8;35(1):0026. doi: 10.34133/csbj.0026 (PMC13082541; doi:10.34133/csbj.0026)
Supplement: Supplementary 1 — Figs. S1 and S2 Tables S1 to S3 Supplementary Data 1 and 2 [file csbj.0026.f1.zip › Supplementary Data_ TCRa sequences.docx]

**Full-length cDNA sequences of selected TCRα**

**EF1 trav7n-6 traj16**

ATGCATTCCTTACATGTTTCACTAGTGTTCCTCTGGCTTCAACTAGGTGGGGTGAGCAGCCAGGAGAAGGTACAGCAGAGCCCAGAATCCCTCATTGTCCCAGAGGGAGCCATGTCCTCCCTCAACTGCACTTTCAGCAACAGTGCTTCTCAGTCCATCTGGTGGTACCAACAGCATCCTGGGAAAGGCCCCGAAGCACTAATATCCATATTCTCTAATGGCAACAAGAAAGAAGGCAGATTGACAGTTTACCTCAATAGAGCCAGCCTGCATGTTTCCCTGCACATCAGAGACTCCCAGCCCAGTGACTCTGCCGTCTACCTCTGTGCAGTGAGGACTTCAAGTGGCCAGAAGCTGGTTTTTGGCCAGGGGACCATATTAAAGGTGTACCTGCACATCCAGAACCCAGAACCTGCTGTGTACCAGTTAAAAGATCCTCGGTCTCAGGACAGCACCCTCTGCCTGTTCACCGACTTTGACTCCCAAATCAATGTGCCGAAAACCATGGAATCTGGAACGTTCATCACTGACAAAACTGTGCTGGACATGAAAGCTATGGATTCCAAGAGCAATGGGGCCATTGCCTGGAGCAACCAGACAAGCTTCACCTGCCAAGATATCTTCAAAGAGACCAACGCCACCTACCCCAGTTCAGACGTTCCCTGTGATGCCACGTTGACTGAGAAAAGCTTTGAAACAGATATGAACCTAAACTTTCAAAACCTGTCAGTTATGGGACTCCGAATCCTCCTGCTGAAAGTAGCCGGATTTAACCTGCTCATGACGCTGAGGCTGTGGTCCAGTTGA

**EF2 trav4n-4 (not trv4-3) traj33**

ATGCAGAGGAACCTGGGAGCTGTGCTGGGGATTCTGTGGGTGCAGATTTGCTGGGTGAGAGGAGATCAGGTGGAGCAGAGTCCTTCAGCCCTGAGCCTCCACGAGGGAACCGGTTCTGCTCTGAGATGCAATTTTACTACCACCATGAGGGCTGTGCAGTGGTTCCGAAAGAATTCCAGGGGCAGCCTCATCAATCTGTTCTACTTGGCTTCAGGAACAAAGGAGAATGGGAGGCTAAAGTCAGCATTTGATTCTAAGGAGCGCTACAGCACCCTGCACATCAGGGATGCCCAGCTGGAGGACTCAGGCACTTACTTCTGTGCTGCTGAGACTAGCAACTATCAGTTGATCTGGGGCTCTGGGACCAAGCTAATTATAAAGCCAGACATCCAGAACCCAGAACCTGCTGTGTACCAGTTAAAAGATCCTCGGTCTCAGGACAGCACCCTCTGCCTGTTCACCGACTTTGACTCCCAAATCAATGTGCCGAAAACCATGGAATCTGGAACGTTCATCACTGACAAAACTGTGCTGGACATGAAAGCTATGGATTCCAAGAGCAATGGGGCCATTGCCTGGAGCAACCAGACAAGCTTCACCTGCCAAGATATCTTCAAAGAGACCAACGCCACCTACCCCAGTTCAGACGTTCCCTGTGATGCCACGTTGACTGAGAAAAGCTTTGAAACAGATATGAACCTAAACTTTCAAAACCTGTCAGTTATGGGACTCCGAATCCTCCTGCTGAAAGTAGCCGGATTTAACCTGCTCATGACGCTGAGGCTGTGGTCCAGTTGA

**EF3 trav14-1 traj18**

ATGGACAAGATTCTGACAGCATCATTTTTACTCCTAGGCCTTCACCTAGCTGGGGTGAATGGCCAGCAGAAGGAGAAACATGACCAGCAGCAGGTGAGACAAAGTCCCCAATCTCTGACAGTCTGGGAAGGAGGAACCACAGTTCTGACCTGCAGTTATGAGGACAGCACTTTTAACTACTTCCCATGGTACCAACAGTTCCCTGGGGAAGGCCCTGCACTTCTGATATCCATACTTTCAGTGTCCGATAAAAAGGAAGATGGACGATTCACAACCTTCTTCAATAAAAGGGAGAAAAAGCTCTCCTTGCACATCATAGACTCTCAGCCTGGAGACTCAGCCACCTACTTCTGTGCAGCAGATAGAGGTTCAGCCTTAGGGAGGCTGCATTTTGGAGCTGGGACTCAGCTGATTGTCATACCTGACATCCAGAACCCAGAACCTGCTGTGTACCAGTTAAAAGATCCTCGGTCTCAGGACAGCACCCTCTGCCTGTTCACCGACTTTGACTCCCAAATCAATGTGCCGAAAACCATGGAATCTGGAACGTTCATCACTGACAAAACTGTGCTGGACATGAAAGCTATGGATTCCAAGAGCAATGGGGCCATTGCCTGGAGCAACCAGACAAGCTTCACCTGCCAAGATATCTTCAAAGAGACCAACGCCACCTACCCCAGTTCAGACGTTCCCTGTGATGCCACGTTGACTGAGAAAAGCTTTGAAACAGATATGAACCTAAACTTTCAAAACCTGTCAGTTATGGGACTCCGAATCCTCCTGCTGAAAGTAGCCGGATTTAACCTGCTCATGACGCTGAGGCTGTGGTCCAGTTGA

**EF4 trav1 traj31**

ATGCTGCAGATGTGGGGGTTTGTTCTCTATCTCTTCCTGACGGTGGGAGGTGCTGCAGGACAGGGTGTGGAGCAGCCTGCCAAATTGATGTCTGTGGAGGGAACCTTTGCTCGGGTCAACTGCACATACAGCACCTCAGGGTTCAACGGGTTATCCTGGTACCAGCAACGTGAAGGCCAAGCCCCTGTATTTCTTTCTTATGTTGTTTTGGATGGTTTGAAGGACAGTGGGCATTTCTCCACTTTCCTGAGCCGCTCGAATGGGTACAGTTACCTGCTTCTGACAGAGCTCCAGATCAAAGACTCTGCCTCATACCTCTGTGCTGCCCTGAATAGCAATAACAGAATCTTCTTTGGTGATGGGACGCAGCTGGTGGTGAAGCCCAACATCCAGAACCCAGAACCTGCTGTGTACCAGTTAAAAGATCCTCGGTCTCAGGACAGCACCCTCTGCCTGTTCACCGACTTTGACTCCCAAATCAATGTGCCGAAAACCATGGAATCTGGAACGTTCATCACTGACAAAACTGTGCTGGACATGAAAGCTATGGATTCCAAGAGCAATGGGGCCATTGCCTGGAGCAACCAGACAAGCTTCACCTGCCAAGATATCTTCAAAGAGACCAACGCCACCTACCCCAGTTCAGACGTTCCCTGTGATGCCACGTTGACTGAGAAAAGCTTTGAAACAGATATGAACCTAAACTTTCAAAACCTGTCAGTTATGGGACTCCGAATCCTCCTGCTGAAAGTAGCCGGATTTAACCTGCTCATGACGCTGAGGCTGTG GTCCAGTTGA

**EF5 trav12-3 (not trav12-1) traj33**

ATGAACATGCGTCCTGTCACCTGCTCAGTTCTTGTGCTCCTCCTAATGCTCAGGAGGAGCAATGGAGATGGAGACTCAGTGACCCAGAAGGAAGGCCTGGTCACTCTCACCGAGGGGTTGCCTGTGATGCTGAACTGCACCTATCAGACTATTTACTCAAATGCTTTCCTTTTCTGGTATGTGCACTATCTCAATGAATCCCCTCGGCTACTCCTGAAGAGCTCCACAGACAACAAGAGGACCGAGCACCAAGGGTTCCACGCCACTCTCCATAAGAGCAGCAGCTCCTTCCATCTGCAGAAGTCCTCAGCGCAGCTGTCAGACTCTGCCCTGTACTACTGTGCTCTGATGGATAGCAACTATCAGTTGATCTGGGGCTCTGGGACCAAGCTAATTATAAAGCCAGACATCCAGAACCCAGAACCTGCTGTGTACCAGTTAAAAGATCCTCGGTCTCAGGACAGCACCCTCTGCCTGTTCACCGACTTTGACTCCCAAATCAATGTGCCGAAAACCATGGAATCTGGAACGTTCATCACTGACAAAACTGTGCTGGACATGAAAGCTATGGATTCCAAGAGCAATGGGGCCATTGCCTGGAGCAACCAGACAAGCTTCACCTGCCAAGATATCTTCAAAGAGACCAACGCCACCTACCCCAGTTCAGACGTTCCCTGTGATGCCACGTTGACTGAGAAAAGCTTTGAAACAGATATGAACCTAAACTTTCAAAACCTGTCAGTTATGGGACTCCGAATCCTCCTGCTGAAAGTAGCCGGATTTAACCTGCTCATGACGCTGAGGCTGTGGTCCAGTTGA

**EF6 trav12-2 (not trav12-1) traj45**

ATGAACATGCGTCCTGTCACCTGCTCAGTTCTTGTGCTCCTCTTAATGCTCAGAAGGAACAATGGAGACTCTGTGACCCAGACAGAAGGCCTGGTCACTCTCACCGAGGGGTTGCCTGTGATGCTGAACTGCACCTATCAGAGTACTTACTCACCTTTCCTTTTCTGGTATGTGCAACATCTCAACGAAGCCCCTAAGCTACTTTTGAAGAGCTTCACAGACAACAAGAGGCCCGAGCACCAAGGGTTCCACGCCACTCTCCATAAGAGCAGCAGCTCCTTCCATCTGCAGAAGTCCTCAGCGCAGCTGTCAGACTCTGCCCTGTACTACTGTCTTACAGAAGGTGCAGATAGACTCACCTTTGGGAAAGGAACTCAGCTGATCATCCAGCCCTACATCCAGAACCCAGAACCTGCTGTGTACCAGTTAAAAGATCCTCGGTCTCAGGACAGCACCCTCTGCCTGTTCACCGACTTTGACTCCCAAATCAATGTGCCGAAAACCATGGAATCTGGAACGTTCATCACTGACAAAACTGTGCTGGACATGAAAGCTATGGATTCCAAGAGCAATGGGGCCATTGCCTGGAGCAACCAGACAAGCTTCACCTGCCAAGATATCTTCAAAGAGACCAACGCCACCTACCCCAGTTCAGACGTTCCCTGTGATGCCACGTTGACTGAGAAAAGCTTTGAAACAGATATGAACCTAAACTTTCAAAACCTGTCAGTTATGGGACTCCGAATCCTCCTGCTGAAAGTAGCCGGATTTAACCTGCTCATGACGCTGAGGCTGTGGTCCAGTTGA

**EF7 trav12-2 (trav12d-2) traj22**

ATGAACATGCGTCCTGACACCTGCTCAGTTCTTGTGCTCCTCTTAATGCTCAGAAGGAACAATGGAGACTCTGTGACCCAGACAGAAGGCCTGGTCACTCTCACCGAGGGGTTGCCTGTGATGCTGAACTGCACCTATCAGAGTACTTACTCACCTTTCCTTTTCTGGTATGTGCAACATCTCAACGAAGCCCCTAAGCTACTTTTGAAGAGCTTCACAGACAACAAGAGGCCCGAGCACCAAGGGTTCCACGCCACTCTCCATAAGAGCAGCAGCTCCTTCCATCTGCAGAAGTCCTCAGCGCAGCTGTCAGACTCTGCCCTGTACTACTGTGCTTTGAGTGAAGCATCTTCTGGCAGCTGGCAACTCATCTTTGGATCTGGAACCCAACTGACAGTTATGCCTGACATCCAGAACCCAGAACCTGCTGTGTACCAGTTAAAAGATCCTCGGTCTCAGGACAGCACCCTCTGCCTGTTCACCGACTTTGACTCCCAAATCAATGTGCCGAAAACCATGGAATCTGGAACGTTCATCACTGACAAAACTGTGCTGGACATGAAAGCTATGGATTCCAAGAGCAATGGGGCCATTGCCTGGAGCAACCAGACAAGCTTCACCTGCCAAGATATCTTCAAAGAGACCAACGCCACCTACCCCAGTTCAGACGTTCCCTGTGATGCCACGTTGACTGAGAAAAGCTTTGAAACAGATATGAACCTAAACTTTCAAAACCTGTCAGTTATGGGACTCCGAATCCTCCTGCTGAAAGTAGCCGGATTTAACCTGCTCATGACGCTGAGGCTGTGGTCCAGTTGA

**EF10 trav14n(d)-2 (not trav14n-3) traj30**

ATGGACAAGATCCTGACAGCATCGTTTTTACTCCTAGGCCTTCACCTAGCTGGGGTGAGTGGCCAGCAGCAGGAGAAACGTGACCAGCAGCAGGTGAGACAAAGTCCCCAATCTCTGACAGTCTGGGAAGGAGAGACAGCAATTCTGAACTGCAGTTATGAGGACAGCACTTTTGACTACTTCCCATGGTACTGGCAGTTCCCTAGGGAAAGCCCTGCACTCCTGATAGCCATACGTCCAGTGTCCAATAAAAAGGAAGATGGACGATTCACAATCTTCTTCAATAAAAGGGAGAAAAAGCTCTCCTTGCACATCACAGACTCTCAGCCTGGAGACTCAGCTACCTACTTCTGTGCAGCAACCCCAAATGCTTACAAAGTCATCTTTGGAAAAGGGACACATCTTCATGTTCTCCCTAACATCCAGAACCCAGAACCTGCTGTGTACCAGTTAAAAGATCCTCGGTCTCAGGACAGCACCCTCTGCCTGTTCACCGACTTTGACTCCCAAATCAATGTGCCGAAAACCATGGAATCTGGAACGTTCATCACTGACAAAACTGTGCTGGACATGAAAGCTATGGATTCCAAGAGCAATGGGGCCATTGCCTGGAGCAACCAGACAAGCTTCACCTGCCAAGATATCTTCAAAGAGACCAACGCCACCTACCCCAGTTCAGACGTTCCCTGTGATGCCACGTTGACTGAGAAAAGCTTTGAAACAGATATGAACCTAAACTTTCAAAACCTGTCAGTTATGGGACTCCGAATCCTCCTGCTGAAAGTAGCCGGATTTAACCTGCTCATGACGCTGAGGCTGTGGTCCAGTTGA

**EF11 trav13-1 (not trav13) traj26**

ATGAAGAGGCTGCTGAGCTCTCTGCTGGGGCTTCTGTGCACCCAGGTTTGCTGGGTGAAAGGACAGCAAGTGCAGCAGAGCCCCGCGTCCTTGGTTCTGCAGGAGGGGGAGAACGCAGAGCTGCAGTGTAACTTTTCCACATCTTTGAACAGTATGCAGTGGTTTTACCAACGTCCTGGGGGAAGTCTCGTCAGCCTGTTCTACAATCCTTCTGGGACAAAGCATAGTGGGAGACTGACATCCACTACAGTCATCAAAGAACGTCGCAGCTCTTTGCACATTTCCTCCTCCCAGACAACAGACTCAGGCACTTATCTCTGTGCTTTTCGAAATAACTATGCCCAGGGATTAACCTTCGGTCTTGGCACCAGAGTATCTGTGTTTCCCTACATCCAGAACCCAGAACCTGCTGTGTACCAGTTAAAAGATCCTCGGTCTCAGGACAGCACCCTCTGCCTGTTCACCGACTTTGACTCCCAAATCAATGTGCCGAAAACCATGGAATCTGGAACGTTCATCACTGACAAAACTGTGCTGGACATGAAAGCTATGGATTCCAAGAGCAATGGGGCCATTGCCTGGAGCAACCAGACAAGCTTCACCTGCCAAGATATCTTCAAAGAGACCAACGCCACCTACCCCAGTTCAGACGTTCCCTGTGATGCCACGTTGACTGAGAAAAGCTTTGAAACAGATATGAACCTAAACTTTCAAAACCTGTCAGTTATGGGACTCCGAATCCTCCTGCTGAAAGTAGCCGGATTTAACCTGCTCATGACGCTGAGGCTGTGGTCCAGTTGA

**EF13 trav10d (not trav10) traj26**

ATGAAGACATCCCTTCACACTGTATTCCTATTCTTGTGGCTGTGGATGGACTGGGAGAGCCATGGAGAGAAGGTCGAGCAACACGAGTCTACACTGAGTGTTCGAGAGGGAGACAGCGCTGTCATCAACTGCACTTACACAGATACTGCCTCATCATACTTCCCTTGGTACAAGCAAGAAGCTGGAAAGAGTCTCCACTTTGTGATAGACATTCGTTCAAATGTGGACAGAAAACAGAGCCAAAGACTTATAGTTTTGTTGGATAAGAAAGCCAAACGATTCTCCCTGCACATCACAGCCACACAGCCTGAAGATTCAGCCATCTACTTCTGTGCAGCAAGCCTTTATGCCCAGGGATTAACCTTCGGTCTTGGCACCAGAGTATCTGTGTTTCCCTACATCCAGAACCCAGAACCTGCTGTGTACCAGTTAAAAGATCCTCGGTCTCAGGACAGCACCCTCTGCCTGTTCACCGACTTTGACTCCCAAATCAATGTGCCGAAAACCATGGAATCTGGAACGTTCATCACTGACAAAACTGTGCTGGACATGAAAGCTATGGATTCCAAGAGCAATGGGGCCATTGCCTGGAGCAACCAGACAAGCTTCACCTGCCAAGATATCTTCAAAGAGACCAACGCCACCTACCCCAGTTCAGACGTTCCCTGTGATGCCACGTTGACTGAGAAAAGCTTTGAAACAGATATGAACCTAAACTTTCAAAACCTGTCAGTTATGGGACTCCGAATCCTCCTGCTGAAAGTAGCCGGATTTAACCTGCTCATGACGCTGAGGCTGTGGTCCAGTTGA

**EF14 trav14-1 traj30**

ATGGACAAGATTCTGACAGCATCATTTTTACTCCTAGGCCTTCACCTAGCTGGGGTGAATGGCCAGCAGAAGGAGAAACATGACCAGCAGCAGGTGAGACAAAGTCCCCAATCTCTGACAGTCTGGGAAGGAGGAACCACAGTTCTGACCTGCAGTTATGAGGACAGCACTTTTAACTACTTCCCATGGTACCAACAGTTCCCTGGGGAAGGCCCTGCACTTCTGATATCCATACTTTCAGTGTCCGATAAAAAGGAAGATGGACGATTCACAACCTTCTTCAATAAAAGGGAGAAAAAGCTCTCCTTGCACATCATAGACTCTCAGCCTGGAGACTCAGCCACCTACTTCTGTGCAGCAGGGGGCACAAATGCTTACAAAGTCATCTTTGGAAAAGGGACACATCTTCATGTTCTCCCTAACATCCAGAACCCAGAACCTGCTGTGTACCAGTTAAAAGATCCTCGGTCTCAGGACAGCACCCTCTGCCTGTTCACCGACTTTGACTCCCAAATCAATGTGCCGAAAACCATGGAATCTGGAACGTTCATCACTGACAAAACTGTGCTGGACATGAAAGCTATGGATTCCAAGAGCAATGGGGCCATTGCCTGGAGCAACCAGACAAGCTTCACCTGCCAAGATATCTTCAAAGAGACCAACGCCACCTACCCCAGTTCAGACGTTCCCTGTGATGCCACGTTGACTGAGAAAAGCTTTGAAACAGATATGAACCTAAACTTTCAAAACCTGTCAGTTATGGGACTCCGAATCCTCCTGCTGAAAGTAGCCGGATTTAACCTGCTCATGACGCTGAGGCTGTGGTCCAGTTGA

**EF15 trav6-2 (not trav6-1) traj21**

ATGAACACTTCTCCAGCTTTAGTGACTGTGATGCTGCTGTTCATGCTTGAGAGGACACATGGAAATTCAGTGACCCAGATGCAAGGTCAAGTGACCCTTTCAGAAGAGGAGTTTCTATTTATAAACTGTACCTATTCAACCACAGGGTACCCGACTCTTTTCTGGTATGTCCAATATCCTGGAGAAGGTCCACAGCTCCTTTTGAAAGTCACAACAGCCAACAATAAGGGAAGCAGCAGAGGTTTTGAAGCTACATATGATAAAGGGACCACGTCCTTCCACTTGCAGAAAGCCTCAGTGCAGGAGTCAGACTCGGCTGTGTACTACTGTGTTCTGGGGATGTCTAATTACAACGTGCTTTACTTCGGATCTGGCACCAAACTCACTGTAGAGCCAAACATCCAGAACCCAGAACCTGCTGTGTACCAGTTAAAAGATCCTCGGTCTCAGGACAGCACCCTCTGCCTGTTCACCGACTTTGACTCCCAAATCAATGTGCCGAAAACCATGGAATCTGGAACGTTCATCACTGACAAAACTGTGCTGGACATGAAAGCTATGGATTCCAAGAGCAATGGGGCCATTGCCTGGAGCAACCAGACAAGCTTCACCTGCCAAGATATCTTCAAAGAGACCAACGCCACCTACCCCAGTTCAGACGTTCCCTGTGATGCCACGTTGACTGAGAAAAGCTTTGAAACAGATATGAACCTAAACTTTCAAAACCTGTCAGTTATGGGACTCCGAATCCTCCTGCTGAAAGTAGCCGGATTTAACCTGCTCATGACGCTGAGGCTGTGGTCCAGTTGA

**EF18 trav12n-3 (not trav12-1) traj26**

ATGCGTCCTGTCACCTGCTCAGTTCTTGTGCTCCTCCTAATGCTCAGGAGGAGCAATGGCGATGGAGACTCCGTGACCCAGACAGAAGGCCTGGTCACTCTCACAGAAGGGTTGCCTGTGATGCTGAACTGCACCTATCAGACTATTTACTCAAATCCTTTCCTTTTCTGGTATGTGCAACATCTCAATGAATCCCCTCGGCTACTCCTGAAGAGCTTCACAGACAACAAGAGGACCGAGCACCAAGGGTTCCACATCACTCTCCATAAGAGCAGCAGCTCCTTCCATCTGCAGAAGTCCTCAGCGCAGCTGTCAGACTCTGCCCTGTACTACTGTGCTCTGAGTGATCGGGGTAACTATGCCCAGGGATTAACCTTCGGTCTTGGCACCAGAGTATCTGTGTTTCCCTACATCCAGAACCCAGAACCTGCTGTGTACCAGTTAAAAGATCCTCGGTCTCAGGACAGCACCCTCTGCCTGTTCACCGACTTTGACTCCCAAATCAATGTGCCGAAAACCATGGAATCTGGAACGTTCATCACTGACAAAACTGTGCTGGACATGAAAGCTATGGATTCCAAGAGCAATGGGGCCATTGCCTGGAGCAACCAGACAAGCTTCACCTGCCAAGATATCTTCAAAGAGACCAACGCCACCTACCCCAGTTCAGACGTTCCCTGTGATGCCACGTTGACTGAGAAAAGCTTTGAAACAGATATGAACCTAAACTTTCAAAACCTGTCAGTTATGGGACTCCGAATCCTCCTGCTGAAAGTAGCCGGATTTAACCTGCTCATGACGCTGAGGCTGTGGTCCAGTTGA

**EF19 trav12-2 traj34**

ATGAACATGCGTCCTGACACCTGCTCAGTTCTTGTGCTCCTCTTAATGCTCAGAAGGAACAATGGAGACTCTGTGACCCAGACAGAAGGCCTGGTCACTCTCACCGAGGGGTTGCCTGTGATGCTGAACTGCACCTATCAGAGTACTTACTCACCTTTCCTTTTCTGGTATGTGCAACATCTCAACGAAGCCCCTAAGCTACTTTTGAAGAGCTTCACAGACAACAAGAGGCCCGAGCACCAAGGGTTCCACGCCACTCTCCATAAGAGCAGCAGCTCCTTCCATCTGCAGAAGTCCTCAGCGCAGCTGTCAGACTCTGCCCTGTACTACTGTGCTTTGCCGTCTTCCAATACCAACAAAGTCGTCTTTGGAACAGGGACCAGATTACAAGTATTACCAAACATCCAGAACCCAGAACCTGCTGTGTACCAGTTAAAAGATCCTCGGTCTCAGGACAGCACCCTCTGCCTGTTCACCGACTTTGACTCCCAAATCAATGTGCCGAAAACCATGGAATCTGGAACGTTCATCACTGACAAAACTGTGCTGGACATGAAAGCTATGGATTCCAAGAGCAATGGGGCCATTGCCTGGAGCAACCAGACAAGCTTCACCTGCCAAGATATCTTCAAAGAGACCAACGCCACCTACCCCAGTTCAGACGTTCCCTGTGATGCCACGTTGACTGAGAAAAGCTTTGAAACAGATATGAACCTAAACTTTCAAAACCTGTCAGTTATGGGACTCCGAATCCTCCTGCTGAAAGTAGCCGGATTTAACCTGCTCATGACGCTGAGGCTGTGGTCCAGTTGA

**EF21 trav12-2 (trav12d-2) traj26**

ATGAACATGCGTCCTGACACCTGCTCAGTTCTTGTGCTCCTCTTAATGCTCAGAAGGAACAATGGAGACTCTGTGACCCAGACAGAAGGCCTGGTCACTCTCACCGAGGGGTTGCCTGTGATGCTGAACTGCACCTATCAGAGTACTTACTCACCTTTCCTTTTCTGGTATGTGCAACATCTCAACGAAGCCCCTAAGCTACTTTTGAAGAGCTTCACAGACAACAAGAGGCCCGAGCACCAAGGGTTCCACGCCACTCTCCATAAGAGCAGCAGCTCCTTCCATCTGCAGAAGTCCTCAGCGCAGCTGTCAGACTCTGCCCTGTACTACTGTGCTTTGAGTGATGGAAACTATGCCCAGGGATTAACCTTCGGTCTTGGCACCAGAGTATCTGTGTTTCCCTACATCCAGAACCCAGAACCTGCTGTGTACCAGTTAAAAGATCCTCGGTCTCAGGACAGCACCCTCTGCCTGTTCACCGACTTTGACTCCCAAATCAATGTGCCGAAAACCATGGAATCTGGAACGTTCATCACTGACAAAACTGTGCTGGACATGAAAGCTATGGATTCCAAGAGCAATGGGGCCATTGCCTGGAGCAACCAGACAAGCTTCACCTGCCAAGATATCTTCAAAGAGACCAACGCCACCTACCCCAGTTCAGACGTTCCCTGTGATGCCACGTTGACTGAGAAAAGCTTTGAAACAGATATGAACCTAAACTTTCAAAACCTGTCAGTTATGGGACTCCGAATCCTCCTGCTGAAAGTAGCCGGATTTAACCTGCTCATGACGCTGAGGCTGTGGTCCAGTTGA

**EF22 trav3d-3 traj30**

ATGAAGACAGTGACTGGACCTTTGTTCCTGTGCTTCTGGCTGCAGCTGAACTGTGTGAGCAGAGGCGAGCAGGTGGAGCAGCGCCCTCCTCACCTGAGTGTCCGGGAGGGAGACAGTGCCGTTATCATCTGCACCTACACAGACCCTAACAGTTATTACTTCTTCTGGTACAAGCAAGAGCCGGGGGCAGGTCTTCAGTTGCTTATGAAGGTTTTCTCAAGTACGGAAATAAACGAAGGACAAGGATTCACTGTCCTACTGAACAAGAAAGACAAACAACTCTCTCTGAACCTCACAGCTGCCCATCCTGGGGACTCAGCCGTGTACTTCTGCGCAGTCAGTGCCACAAATGCTTACAAAGTCATCTTTGGAAAAGGGACACATCTTCATGTTCTCCCTAACATCCAGAACCCAGAACCTGCTGTGTACCAGTTAAAAGATCCTCGGTCTCAGGACAGCACCCTCTGCCTGTTCACCGACTTTGACTCCCAAATCAATGTGCCGAAAACCATGGAATCTGGAACGTTCATCACTGACAAAACTGTGCTGGACATGAAAGCTATGGATTCCAAGAGCAATGGGGCCATTGCCTGGAGCAACCAGACAAGCTTCACCTGCCAAGATATCTTCAAAGAGACCAACGCCACCTACCCCAGTTCAGACGTTCCCTGTGATGCCACGTTGACTGAGAAAAGCTTTGAAACAGATATGAACCTAAACTTTCAAAACCTGTCAGTTATGGGACTCCGAATCCTCCTGCTGAAAGTAGCCGGATTTAACCTGCTCATGACGCTGAGGCTGTGGTCCAGTTGA

**EF26 trav12-3 traj13**

ATGCGTCCTGGCACCTGCTCAGTTCTTGTGCTCCTCCTAATGCTCAGGAGGAGCAATGGAGATGGAGACTCAGTGACCCAGAAGGAAGGCCTGGTCACTCTCACCGAGGGGTTGCCTGTGATGCTGAACTGCACCTATCAGACTATTTACTCAAATGCTTTCCTTTTCTGGTATGTGCACTATCTCAATGAATCCCCTCGGCTACTCCTGAAGAGCTCCACAGACAACAAGAGGACCGAGCACCAAGGGTTCCACGCCACTCTCCATAAGAGCAGCAGCTCCTTCCATCTGCAGAAGTCCTCAGCGCAGCTGTCAGACTCTGCCCTGTACTACTGTGCTCTGCCAAATTCTGGGACTTACCAGAGGTTTGGAACTGGGACAAAACTCCAAGTCGTTCCAAACATCCAGAACCCAGAACCTGCTGTGTACCAGTTAAAAGATCCTCGGTCTCAGGACAGCACCCTCTGCCTGTTCACCGACTTTGACTCCCAAATCAATGTGCCGAAAACCATGGAATCTGGAACGTTCATCACTGACAAAACTGTGCTGGACATGAAAGCTATGGATTCCAAGAGCAATGGGGCCATTGCCTGGAGCAACCAGACAAGCTTCACCTGCCAAGATATCTTCAAAGAGACCAACGCCACCTACCCCAGTTCAGACGTTCCCTGTGATGCCACGTTGACTGAGAAAAGCTTTGAAACAGATATGAACCTAAACTTTCAAAACCTGTCAGTTATGGGACTCCGAATCCTCCTGCTGAAAGTAGCCGGATTTAACCTGCTCATGACGCTGAGGCTGTGGTCCAGTTGA

**EF27 trav12-3 traj33**

ATGCGTCCTGGCACCTGCTCAGTTCTTGTGCTCCTCCTAATGCTCAGGAGGAGCAATGGAGATGGAGACTCAGTGACCCAGAAGGAAGGCCTGGTCACTCTCACCGAGGGGTTGCCTGTGATGCTGAACTGCACCTATCAGACTATTTACTCAAATGCTTTCCTTTTCTGGTATGTGCACTATCTCAATGAATCCCCTCGGCTACTCCTGAAGAGCTCCACAGACAACAAGAGGACCGAGCACCAAGGGTTCCACGCCACTCTCCATAAGAGCAGCAGCTCCTTCCATCTGCAGAAGTCCTCAGCGCAGCTGTCAGACTCTGCCCTGTACTACTGTGCTCTGGGGGATAGCAACTATCAGTTGATCTGGGGCTCTGGGACCAAGCTAATTATAAAGCCAGACATCCAGAACCCAGAACCTGCTGTGTACCAGTTAAAAGATCCTCGGTCTCAGGACAGCACCCTCTGCCTGTTCACCGACTTTGACTCCCAAATCAATGTGCCGAAAACCATGGAATCTGGAACGTTCATCACTGACAAAACTGTGCTGGACATGAAAGCTATGGATTCCAAGAGCAATGGGGCCATTGCCTGGAGCAACCAGACAAGCTTCACCTGCCAAGATATCTTCAAAGAGACCAACGCCACCTACCCCAGTTCAGACGTTCCCTGTGATGCCACGTTGACTGAGAAAAGCTTTGAAACAGATATGAACCTAAACTTTCAAAACCTGTCAGTTATGGGACTCCGAATCCTCCTGCTGAAAGTAGCCGGATTTAACCTGCTCATGACGCTGAGGCTGTGGTCCAGTTGA

**EF29 trav7-2 traj22**

ATGAAATCCTTTAGTATTTCCCTAGTGGTCCTGTGGCTTCAGCTAAACTGGGTGAACAGCCAACAGAAGGTGCAGCAGAGCCCAGAATCCCTCATTGTTCCAGAGGGAGGCATGGCCTCTCTCAACTGCACTTCCAGTGATCGTAATGTTGACTACTTCTGGTGGTACAGACAGCACTCTGGGAAAAGCCCCAAGATGCTGATGTCTATCTTCTCCAATGGTGAAAAGGAAGAAGGCAGATTCACAGTTCACCTCAATAAAGCCAGCCTGCATACTTCCCTGCACATCAGAGACTCCCAGCCCAGTGACTCTGCTCTCTACCTCTGTGCAGCCGCATCTTCTGGCAGCTGGCAACTCATCTTTGGATCTGGAACCCAACTGACAGTTATGCCTGACATCCAGAACCCAGAACCTGCTGTGTACCAGTTAAAAGATCCTCGGTCTCAGGACAGCACCCTCTGCCTGTTCACCGACTTTGACTCCCAAATCAATGTGCCGAAAACCATGGAATCTGGAACGTTCATCACTGACAAAACTGTGCTGGACATGAAAGCTATGGATTCCAAGAGCAATGGGGCCATTGCCTGGAGCAACCAGACAAGCTTCACCTGCCAAGATATCTTCAAAGAGACCAACGCCACCTACCCCAGTTCAGACGTTCCCTGTGATGCCACGTTGACTGAGAAAAGCTTTGAAACAGATATGAACCTAAACTTTCAAAACCTGTCAGTTATGGGACTCCGAATCCTCCTGCTGAAAGTAGCCGGATTTAACCTGCTCATGACGCTGAGGCTGTGGTCCAGTTGA

**EF30 trav12-2 traj40**

ATGAACATGCGTCCTGACACCTGCTCAGTTCTTGTGCTCCTCTTAATGCTCAGAAGGAACAATGGAGACTCTGTGACCCAGACAGAAGGCCTGGTCACTCTCACCGAGGGGTTGCCTGTGATGCTGAACTGCACCTATCAGAGTACTTACTCACCTTTCCTTTTCTGGTATGTGCAACATCTCAACGAAGCCCCTAAGCTACTTTTGAAGAGCTTCACAGACAACAAGAGGCCCGAGCACCAAGGGTTCCACGCCACTCTCCATAAGAGCAGCAGCTCCTTCCATCTGCAGAAGTCCTCAGCGCAGCTGTCAGACTCTGCCCTGTACTACTGTGCTTTGAGTGAGAGGACAGGAAACTACAAATACGTCTTTGGAGCAGGTACCAGACTGAAGGTTATAGCACACATCCAGAACCCAGAACCTGCTGTGTACCAGTTAAAAGATCCTCGGTCTCAGGACAGCACCCTCTGCCTGTTCACCGACTTTGACTCCCAAATCAATGTGCCGAAAACCATGGAATCTGGAACGTTCATCACTGACAAAACTGTGCTGGACATGAAAGCTATGGATTCCAAGAGCAATGGGGCCATTGCCTGGAGCAACCAGACAAGCTTCACCTGCCAAGATATCTTCAAAGAGACCAACGCCACCTACCCCAGTTCAGACGTTCCCTGTGATGCCACGTTGACTGAGAAAAGCTTTGAAACAGATATGAACCTAAACTTTCAAAACCTGTCAGTTATGGGACTCCGAATCCTCCTGCTGAAAGTAGCCGGATTTAACCTGCTCATGACGCTGAGGCTGTGGTCCAGTTGA

**EM1 trav7-5 traj40**

ATGAAATCTTTGAGTGTTTCACTAGTGGTCCTGTGGCTCCAGTTTAATTGGGTGAGCAGCCAGCAGAAGGTGCAGCAGAGCCCAGAATCCCTCACTGTCTCAGAGGGAGCCATGGCCTCTCTCAACTGCACGTTCAGTGATGGTACTTCTAACAACTTCAGGTGGTACAGACAGCATTCTGCGAAAGGCCTTGAGGTGCTAGTGTCCATCTTCTCTGATGGTGAAAAGGAAGAAGGCAGATTTACAGCTCACCTCAATAGAGCCAACTTGCATGTTTCCCTACACATCAGAGAACCACAACCCAGTGACTCTGCTGTCTACCTCTGTGCAGTGAGGGGAGGAAACTACAAATACGTCTTTGGAGCAGGTACCAGACTGAAGGTTATAGCACACATCCAGAACCCAGAACCTGCTGTGTACCAGTTAAAAGATCCTCGGTCTCAGGACAGCACCCTCTGCCTGTTCACCGACTTTGACTCCCAAATCAATGTGCCGAAAACCATGGAATCTGGAACGTTCATCACTGACAAAACTGTGCTGGACATGAAAGCTATGGATTCCAAGAGCAATGGGGCCATTGCCTGGAGCAACCAGACAAGCTTCACCTGCCAAGATATCTTCAAAGAGACCAACGCCACCTACCCCAGTTCAGACGTTCCCTGTGATGCCACGTTGACTGAGAAAAGCTTTGAAACAGATATGAACCTAAACTTTCAAAACCTGTCAGTTATGGGACTCCGAATCCTCCTGCTGAAAGTAGCCGGATTTAACCTGCTCATGACGCTGAGGCTGTGGTCCAGTTGA

**EM2 trav7-4 traj39**

ATGAAATCCTTGAGTGTTTCACTAGTGGTCCTGTGGCTCCAGTTAAACTGCGTGAGGAGCCAGCAGAAGGTGCAGCAGAGCCCAGAATCCCTCAGTGTCCCAGAGGGAGGCATGGCCTCTCTCAACTGCACTTCAAGTGATCGTAATTTTCAGTACTTCTGGTGGTACAGACAGCATTCTGGAGAAGGCCCCAAGGCACTGATGTCAATCTTCTCTGATGGTGACAAGAAAGAAGGCAGATTCACAGCTCACCTCAATAAGGCCAGCCTGCATGTTTCCCTGCACATCAGAGACTCCCAGCCCAGTGACTCCGCTCTCTACTTCTGTGCAGCTAGTGAGCTCGCAGGTGCCAAGCTCACATTCGGAGGGGGAACAAGGTTAACGGTCAGACCCGACATCCAGAACCCAGAACCTGCTGTGTACCAGTTAAAAGATCCTCGGTCTCAGGACAGCACCCTCTGCCTGTTCACCGACTTTGACTCCCAAATCAATGTGCCGAAAACCATGGAATCTGGAACGTTCATCACTGACAAAACTGTGCTGGACATGAAAGCTATGGATTCCAAGAGCAATGGGGCCATTGCCTGGAGCAACCAGACAAGCTTCACCTGCCAAGATATCTTCAAAGAGACCAACGCCACCTACCCCAGTTCAGACGTTCCCTGTGATGCCACGTTGACTGAGAAAAGCTTTGAAACAGATATGAACCTAAACTTTCAAAACCTGTCAGTTATGGGACTCCGAATCCTCCTGCTGAAAGTAGCCGGATTTAACCTGCTCATGACGCTGAGGCTGTGGTCCAGTTGA

**EM3 trav21/dv12 traj58**

ATGGGATGTGTGAGTGGAATTGCCATTCTCCTGGCTTTGGGTATTGCGGGTGATGCTAAGACTACACAACCAGATTCAATGGAAAGTACTGAAGGAGAAACGGTGCACTTGCCTTGTAGCCACGCCACAATCAGTGGAAACGAGTACATCTATTGGTACCGACAGGTTCCTCTTCAGGGTCCAGAATATGTGACTCACGGTCTACAACAAAATACAACCAATAGTATGGCTTTCCTGGCTATTGCCTCTGACAGAAAGTCAAGCACCTTGATCCTGCCTCATGTCAGCCTGAGAGACGCGGCTGTGTACCACTGTATCCTGAGAGTCCCCCCTCAGCAAGGCACTGGGTCTAAGCTGTCATTTGGGAAGGGGGCAAAGCTCACAGTGAGTCCAGACATCCAGAACCCAGAACCTGCTGTGTACCAGTTAAAAGATCCTCGGTCTCAGGACAGCACCCTCTGCCTGTTCACCGACTTTGACTCCCAAATCAATGTGCCGAAAACCATGGAATCTGGAACGTTCATCACTGACAAAACTGTGCTGGACATGAAAGCTATGGATTCCAAGAGCAATGGGGCCATTGCCTGGAGCAACCAGACAAGCTTCACCTGCCAAGATATCTTCAAAGAGACCAACGCCACCTACCCCAGTTCAGACGTTCCCTGTGATGCCACGTTGACTGAGAAAAGCTTTGAAACAGATATGAACCTAAACTTTCAAAACCTGTCAGTTATGGGACTCCGAATCCTCCTGCTGAAAGTAGCCGGATTTAACCTGCTCATGACGCTGAGGCTGTGGTCCAGTTGA

**EM4 trav4d-3 Traj39**

ATGGAGAGGAACCTGGGAGCTGTGCTGGGGATTCTGTGGGTGCAGATTTGCTGGGTGAGCGGAGATAAGGTGAAGCAAAGTCCCTCAGCGCTGAGTCTCCAAGAGGGAACCAATTCTGCTCTGAGATGCAATTTTTCTATCGCTGCGACAACTGTGCAGTGGTTCCTACAGAATCCCAGGGGCAGCCTCATGAATCTTTTTTACCTGGTGCCAGGAACAAAGGAGAATGGGAGGTTAAAGTCAGCATTTGATTCTAAGGAGAGCTACAGCACCCTGCACATTAGGGATGCCCAGCTGGAGGACTCAGGCACTTACTTCTGTGCTAGGAATGCAGGTGCCAAGCTCACATTCGGAGGGGGAACAAGGTTAACGGTCAGACCCGACATCCAGAACCCAGAACCTGCTGTGTACCAGTTAAAAGATCCTCGGTCTCAGGACAGCACCCTCTGCCTGTTCACCGACTTTGACTCCCAAATCAATGTGCCGAAAACCATGGAATCTGGAACGTTCATCACTGACAAAACTGTGCTGGACATGAAAGCTATGGATTCCAAGAGCAATGGGGCCATTGCCTGGAGCAACCAGACAAGCTTCACCTGCCAAGATATCTTCAAAGAGACCAACGCCACCTACCCCAGTTCAGACGTTCCCTGTGATGCCACGTTGACTGAGAAAAGCTTTGAAACAGATATGAACCTAAACTTTCAAAACCTGTCAGTTATGGGACTCCGAATCCTCCTGCTGAAAGTAGCCGGATTTAACCTGCTCATGACGCTGAGGCTGTGGTCCAGTTGA

**EM5 trav16n (not trav 16d-dv1) traj12**

ATGCTGATTCTAAGCCTGTTGGGAGCAGCCTTTGGCTCCATTTGTTTTGCAGCAACCAGCATGGCCCAGAAGGTAACACAGACTCAGACTTCAATTTCTGTGGTGGAGAAGACAACGGTGACAATGGACTGTGTGTATGAAACCCGGGACAGTTCTTACTTCTTATTCTGGTACAAGCAAACAGCAAGTGGGGAAATAGTTTTCCTTATTCGTCAGGACTCTTACAAAAAGGAAAATGCAACAGTGGGTCATTATTCTCTGAACTTTCAGAAGCCAAAAAGTTCCATCGGACTCATCATCACCGCCACACAGATTGAGGACTCAGCAGTATATTTCTGTGCTATGAGAGGGACTGGAGGCTATAAAGTGGTCTTTGGAAGTGGGACTCGATTGCTGGTAAGCCCTGACATCCAGAACCCAGAACCTGCTGTGTACCAGTTAAAAGATCCTCGGTCTCAGGACAGCACCCTCTGCCTGTTCACCGACTTTGACTCCCAAATCAATGTGCCGAAAACCATGGAATCTGGAACGTTCATCACTGACAAAACTGTGCTGGACATGAAAGCTATGGATTCCAAGAGCAATGGGGCCATTGCCTGGAGCAACCAGACAAGCTTCACCTGCCAAGATATCTTCAAAGAGACCAACGCCACCTACCCCAGTTCAGACGTTCCCTGTGATGCCACGTTGACTGAGAAAAGCTTTGAAACAGATATGAACCTAAACTTTCAAAACCTGTCAGTTATGGGACTCCGAATCCTCCTGCTGAAAGTAGCCGGATTTAACCTGCTCATGACGCTGAGGCTGTGGTCCAGTTGA

**EM6 trav7-4 (not trav 7n-4) traj42**

ATGAAATCCTTGAGTGTTTCACTAGTGGTCCTGTGGCTCCAGTTAAACTGCGTGAGGAGCCAGCAGAAGGTGCAGCAGAGCCCAGAATCCCTCAGTGTCCCAGAGGGAGGCATGGCCTCTCTCAACTGCACTTCAAGTGATCGTAATTTTCAGTACTTCTGGTGGTACAGACAGCATTCTGGAGAAGGCCCCAAGGCACTGATGTCAATCTTCTCTGATGGTGACAAGAAAGAAGGCAGATTCACAGCTCACCTCAATAAGGCCAGCCTGCATGTTTCCCTGCACATCAGAGACTCCCAGCCCAGTGACTCCGCTCTCTACTTCTGTGCAGTTAGGAATTCTGGAGGAAGCAATGCAAAGCTAACCTTCGGGAAAGGCACTAAACTCTCTGTTAAATCAAACATCCAGAACCCAGAACCTGCTGTGTACCAGTTAAAAGATCCTCGGTCTCAGGACAGCACCCTCTGCCTGTTCACCGACTTTGACTCCCAAATCAATGTGCCGAAAACCATGGAATCTGGAACGTTCATCACTGACAAAACTGTGCTGGACATGAAAGCTATGGATTCCAAGAGCAATGGGGCCATTGCCTGGAGCAACCAGACAAGCTTCACCTGCCAAGATATCTTCAAAGAGACCAACGCCACCTACCCCAGTTCAGACGTTCCCTGTGATGCCACGTTGACTGAGAAAAGCTTTGAAACAGATATGAACCTAAACTTTCAAAACCTGTCAGTTATGGGACTCCGAATCCTCCTGCTGAAAGTAGCCGGATTTAACCTGCTCATGACGCTGAGGCTGTGGTCCAGTTGA

**EM7 trav6n-7 (not trav 6-6) traj31**

ATGGGTCTAAAGATGAACTCTTCTCCAGGCTTCATGACTGTGATGCTCCTCATATTCACAAGGGCCCATGGAGACTCAGTGACTCAGACGGAAGGTCAAGTGGCCCTCTCAGAAGAGGACTTTCTTACGATACACTGCAACTACTCAGCCTCAGGGTACCCAGCTCTGTTCTGGTATGTGCAGTATCCCGGAGAAGGTCCACAGTTCCTCTTTAGAGCCTCAAGGGACAAAGAGAAAGGAAGCAGCAGAGGTTTTGAAGCTACATATGATAAAGGGACCACCTCCTTCCACTTGCGGAAAGCCTCAGTGCAAGAGTCAGACTCGGCTGTGTACTACTGTGCTCTGGGTGTTAGCAATAACAGAATCTTCTTTGGTGATGGGACGCAGCTGGTGGTGAAGCCCAACATCCAGAACCCAGAACCTGCTGTGTACCAGTTAAAAGATCCTCGGTCTCAGGACAGCACCCTCTGCCTGTTCACCGACTTTGACTCCCAAATCAATGTGCCGAAAACCATGGAATCTGGAACGTTCATCACTGACAAAACTGTGCTGGACATGAAAGCTATGGATTCCAAGAGCAATGGGGCCATTGCCTGGAGCAACCAGACAAGCTTCACCTGCCAAGATATCTTCAAAGAGACCAACGCCACCTACCCCAGTTCAGACGTTCCCTGTGATGCCACGTTGACTGAGAAAAGCTTTGAAACAGATATGAACCTAAACTTTCAAAACCTGTCAGTTATGGGACTCCGAATCCTCCTGCTGAAAGTAGCCGGATTTAACCTGCTCATGACGCTGAGGCTGTGGTCCAGTTGA

**EM8 trav12n/d3 (not trav 12-1) traj32**

ATGAACATGCGTCCTGTCACCTGCTCAGTTCTTGTGCTCCTCCTAATGCTCAGGAGGAGCAATGGCGATGGAGACTCCGTGACCCAGACAGAAGGCCTGGTCACTCTCACAGAAGGGTTGCCTGTGATGCTGAACTGCACCTATCAGACTATTTACTCAAATCCTTTCCTTTTCTGGTATGTGCAACATCTCAATGAATCCCCTCGGCTACTCCTGAAGAGCTTCACAGACAACAAGAGGACCGAGCACCAAGGGTTCCACGCCACTCTCCATAAGAGCAGCAGCTCCTTCCATCTGCAGAAGTCCTCAGCGCAGCTGTCAGACTCTGCCCTGTACTACTGTGCTCTGAGGGGGAGCAGTGGCAACAAGCTCATCTTTGGAATTGGGACTCTGCTTTCTGTCAAGCCAAACATCCAGAACCCAGAACCTGCTGTGTACCAGTTAAAAGATCCTCGGTCTCAGGACAGCACCCTCTGCCTGTTCACCGACTTTGACTCCCAAATCAATGTGCCGAAAACCATGGAATCTGGAACGTTCATCACTGACAAAACTGTGCTGGACATGAAAGCTATGGATTCCAAGAGCAATGGGGCCATTGCCTGGAGCAACCAGACAAGCTTCACCTGCCAAGATATCTTCAAAGAGACCAACGCCACCTACCCCAGTTCAGACGTTCCCTGTGATGCCACGTTGACTGAGAAAAGCTTTGAAACAGATATGAACCTAAACTTTCAAAACCTGTCAGTTATGGGACTCCGAATCCTCCTGCTGAAAGTAGCCGGATTTAACCTGCTCATGACGCTGAGGCTGTGGTCCAGTTGA

**EM9 trav4d-4 traj18**

ATGCAGAGGAACCTGGGAGCTGTGCTGGGGATTCTGTGGGTGCAGATTTGCTGGGTGAGAGGAGATCAGGTGGAGCAGAGTCCTTCAGCCСTGAGCCTCCACGAGGGAACCGGTTCTGCTCTGAGATGCAATTTTACTACCACCATGAGGGCTGTGCAGTGGTTCCGAAAGAATTCCAGGGGCAGCCTCATCAATCTGTTCTACTTGGCTTCAGGAACAAAGGAGAATGGGAGGCTAAAGTCAGCATTTGATTCTAAGGAGCGCTACAGCACCCTGCACATCAGGGATGCCCAGCTGGAGGACTCAGGCACTTACTTCTGTGCTGCTAGAGGTTCAGCCTTAGGGAGGCTGCATTTTGGAGCTGGGACTCAGCTGATTGTCATACCTGACATCCAGAACCCAGAACCTGCTGTGTACCAGTTAAAAGATCCTCGGTCTCAGGACAGCACCCTCTGCCTGTTCACCGACTTTGACTCCCAAATCAATGTGCCGAAAACCATGGAATCTGGAACGTTCATCACTGACAAAACTGTGCTGGACATGAAAGCTATGGATTCCAAGAGCAATGGGGCCATTGCCTGGAGCAACCAGACAAGCTTCACCTGCCAAGATATCTTCAAAGAGACCAACGCCACCTACCCCAGTTCAGACGTTCCCTGTGATGCCACGTTGACTGAGAAAAGCTTTGAAACAGATATGAACCTAAACTTTCAAAACCTGTCAGTTATGGGACTCCGAATCCTCCTGCTGAAAGTAGCCGGATTTAACCTGCTCATGACGCTGAGGCTGTGGTCCAGTTGA

**EM10 trav7-5 traj17**

ATGAAATCCTTGAGTGTTTCACTAGTGGTCCTGTGGCTCCAGTTTAATTGGGTGAGCAGCCAGCAGAAGGTGCAGCAGAGCCCAGAATCCCTCACTGTCTCAGAGGGAGCCATGGCCTCTCTCAACTGCACGTTCAGTGATGGTACTTCTAACAACTTCAGGTGGTACAGACAGCATTCTGCGAAAGGCCTTGAGGTGCTAGTGTCCATCTTCTCTGATGGTGAAAAGGAAGAAGGCAGATTTACAGCTCACCTCAATAGAGCCAACTTGCATGTTTCCCTACACATCAGAGAACCACAACCCAGTGACTCTGCTGTCTACCTCTGTGCAGTGAGGAACAGTGCAGGGAACAAGCTAACTTTTGGAATCGGAACCAGGGTGCTGGTCAGGCCAGACATCCAGAACCCAGAACCTGCTGTGTACCAGTTAAAAGATCCTCGGTCTCAGGACAGCACCCTCTGCCTGTTCACCGACTTTGACTCCCAAATCAATGTGCCGAAAACCATGGAATCTGGAACGTTCATCACTGACAAAACTGTGCTGGACATGAAAGCTATGGATTCCAAGAGCAATGGGGCCATTGCCTGGAGCAACCAGACAAGCTTCACCTGCCAAGATATCTTCAAAGAGACCAACGCCACCTACCCCAGTTCAGACGTTCCCTGTGATGCCACGTTGACTGAGAAAAGCTTTGAAACAGATATGAACCTAAACTTTCAAAACCTGTCAGTTATGGGACTCCGAATCCTCCTGCTGAAAGTAGCCGGATTTAACCTGCTCATGACGCTGAGGCTGTGGTCCAGTTGA

**EM12 trav6-2 traj22**

ATGAACACTTCTCCAGCTTTAGTGACTGTGATGCTGCTGTTCATGCTTGAGAGGACACATGGAAATTCAGTGACCCAGATGCAAGGTCAAGTGACCCTTTCAGAAGAGGAGTTTCTATTTATAAACTGTACTTATTCAACCACATGGTACCCGACTCTTTTCTGGTATGTCCAATATCCTGGAGAAGGTCCACAGCTCCTGTTGAAAGTCACAACAGCCAACAATAAGGGAAGCAGCAGAGGTTTTGAAGCTACATATGATAAAGGAACAACGTCCTTCCACTTGCAGAAAGCCTCAGTGCAGGAGTCAGACTCGGCTGTGTACTACTGTGTTCTGGGTGATCAGTCTTCTGGCAGCTGGCAACTCATCTTTGGATCTGGAACCCAACTGACAGTTATGCCTGACATCCAGAACCCAGAACCTGCTGTGTACCAGTTAAAAGATCCTCGGTCTCAGGACAGCACCCTCTGCCTGTTCACCGACTTTGACTCCCAAATCAATGTGCCGAAAACCATGGAATCTGGAACGTTCATCACTGACAAAACTGTGCTGGACATGAAAGCTATGGATTCCAAGAGCAATGGGGCCATTGCCTGGAGCAACCAGACAAGCTTCACCTGCCAAGATATCTTCAAAGAGACCAACGCCACCTACCCCAGTTCAGACGTTCCCTGTGATGCCACGTTGACTGAGAAAAGCTTTGAAACAGATATGAACCTAAACTTTCAAAACCTGTCAGTTATGGGACTCCGAATCCTCCTGCTGAAAGTAGCCGGATTTAACCTGCTCATGACGCTGAGGCTGTGGTCCAGTTGA

**EM13 trav8-1 traj43**

ATGCACAGCCTCCTGGGGTTGTTGTTGTGGCTGCAACTGACAAGGGTGAATAGTCAACTAGCAGAAGAGAATTCGTGGGCCCTGAGCGTCCACGAGGGTGAAAGTGTCACGGTGAATTGTAGTTACAAGACATCCATAACTGCCCTACAGTGGTACAGACAGAAGTCAGGCAAAGGCCCTGCCCAACTAATCTTAATACGTTCAAATGAGAGAGAGAAGCGCAATGGAAGACTCAGAGCCACCCTTGACACCTCCAGCCAGAGCAGCTCCTTGTCCATCACTGCTACTCGGTGTGAAGACACCGCTGTGTACTTCTGTGCTATGAGGCCGGATAACAACAATGCCCCACGATTTGGAGCGGGAACCAAATTATCAGTAAAACCAAACATCCAGAACCCAGAACCTGCTGTGTACCAGTTAAAAGATCCTCGGTCTCAGGACAGCACCCTCTGCCTGTTCACCGACTTTGACTCCCAAATCAATGTGCCGAAAACCATGGAATCTGGAACGTTCATCACTGACAAAACTGTGCTGGACATGAAAGCTATGGATTCCAAGAGCAATGGGGCCATTGCCTGGAGCAACCAGACAAGCTTCACCTGCCAAGATATCTTCAAAGAGACCAACGCCACCTACCCCAGTTCAGACGTTCCCTGTGATGCCACGTTGACTGAGAAAAGCTTTGAAACAGATATGAACCTAAACTTTCAAAACCTGTCAGTTATGGGACTCCGAATCCTCCTGCTGAAAGTAGCCGGATTTAACCTGCTCATGACGCTGAGGCTGTGGTCCAGTTGA

**EM15 trav12n3 (not trav 12-1) traj18**

ATGCGTCCTGTCACCTGCTCAGTTCTTGTGCTCCTcCTAATGCTCAGGAGGAGCAATGGCGATGGAGACTCCGTGACCCAGACAGAAGGCCTGGTCACTCTCACAGAAGGGTTGCCTGTGATGCTGAACTGCACCTATCAGACTATTTACTCAAATCCTTTCCTTTTCTGGTATGTGCAACATCTCAATGAATCCCCTCGGCTACTCCTGAAGAGCTTCACAGACAACAAGAGGACCGAGCACCAAGGGTTCCACGCCACTCTCCATAAGAGCAGCAGCTCCTTCCATCTGCAGAAGTCCTCAGCGCAGCTGTCAGACTCTGCCCTGTACTACTGTGCTCTGAGTGGAAGAGGTTCAGCCTTAGGGAGGCTGCATTTTGGAGCTGGGACTCAGCTGATTGTCATACCTGACATCCAGAACCCAGAACCTGCTGTGTACCAGTTAAAAGATCCTCGGTCTCAGGACAGCACCCTCTGCCTGTTCACCGACTTTGACTCCCAAATCAATGTGCCGAAAACCATGGAATCTGGAACGTTCATCACTGACAAAACTGTGCTGGACATGAAAGCTATGGATTCCAAGAGCAATGGGGCCATTGCCTGGAGCAACCAGACAAGCTTCACCTGCCAAGATATCTTCAAAGAGACCAACGCCACCTACCCCAGTTCAGACGTTCCCTGTGATGCCACGTTGACTGAGAAAAGCTTTGAAACAGATATGAACCTAAACTTTCAAAACCTGTCAGTTATGGGACTCCGAATCCTCCTGCTGAAAGTAGCCGGATTTAACCTGCTCATGACGCTGAGGCTGTGGTCCAGTTGA

**EM17 trav16n traj33**

ATGCTGATTCTAAGCCTGTTGGGAGCAGCCTTTGGCTCCATTTGTTTTGCAGCAACCAGCATGGCCCAGAAGGTAACACAGACTCAGACTTCAATTTCTGTGGTGGAGAAGACAACGGTGACAATGGACTGTGTGTATGAAACCCGGGACAGTTCTTACTTCTTATTCTGGTACAAGCAAACAGCAAGTGGGGAAATAGTTTTCCTTATTCGTCAGGACTCTTACAAAAAGGAAAATGCAACAGTGGGTCATTATTCTCTGAACTTTCAGAAGCCAAAAAGTTCCATCGGACTCATCATCACCGCCACACAGATTGAGGACTCAGCAGTATATTTCTGTGCTATGAGAGGGATGGATAGCAACTATCAGTTGATCTGGGGCTCTGGGACCAAGCTAATTATAAAGCCAGACATCCAGAACCCAGAACCTGCTGTGTACCAGTTAAAAGATCCTCGGTCTCAGGACAGCACCCTCTGCCTGTTCACCGACTTTGACTCCCAAATCAATGTGCCGAAAACCATGGAATCTGGAACGTTCATCACTGACAAAACTGTGCTGGACATGAAAGCTATGGATTCCAAGAGCAATGGGGCCATTGCCTGGAGCAACCAGACAAGCTTCACCTGCCAAGATATCTTCAAAGAGACCAACGCCACCTACCCCAGTTCAGACGTTCCCTGTGATGCCACGTTGACTGAGAAAAGCTTTGAAACAGATATGAACCTAAACTTTCAAAACCTGTCAGTTATGGGACTCCGAATCCTCCTGCTGAAAGTAGCCGGATTTAACCTGCTCATGACGCTGAGGCTGTGGTCCAGTTGA

**EM18 trav16n (not trav 16d-dv11) traj17**

ATGCTGATTCTAAGCCTGTTGGGAGCAGCCTTTGGATCCATTTGTTTTGCAGCAACCAGCATGGCCCAGAAGGTAACACAGACTCAGACTTCAATTTCTGTGGTGGAGAAGACAACGGTGACAATGGACTGTGTGTATGAAACCCGGGACAGTTCTTACTTCTTATTCTGGTACAAGCAAACAGCAAGTGGGGAAATAGTTTTCCTTATTCGTCAGGACTCTTACAAAAAGGAAAATGCAACAGTGGGTCATTATTCTCTGAACTTTCAGAAGCCAAAAAGTTCCATCGGACTCATCATCACCGCCACACAGATTGAGGACTCAGCAGTATATTTCTGTGCTATGAGGATGACTAACAGTGCAGGGAACAAGCTAACTTTTGGAATCGGAACCAGGGTGCTGGTCAGGCCAGACATCCAGAACCCAGAACCTGCTGTGTACCAGTTAAAAGATCCTCGGTCTCAGGACAGCACCCTCTGCCTGTTCACCGACTTTGACTCCCAAATCAATGTGCCGAAAACCATGGAATCTGGAACGTTCATCACTGACAAAACTGTGCTGGACATGAAAGCTATGGATTCCAAGAGCAATGGGGCCATTGCCTGGAGCAACCAGACAAGCTTCACCTGCCAAGATATCTTCAAAGAGACCAACGCCACCTACCCCAGTTCAGACGTTCCCTGTGATGCCACGTTGACTGAGAAAAGCTTTGAAACAGATATGAACCTAAACTTTCAAAACCTGTCAGTTATGGGACTCCGAATCCTCCTGCTGAAAGTAGCCGGATTTAACCTGCTCATGACGCTGAGGCTGTGGTCCAGTTGA

**EM19 trav7-5 (not trav7d-5) traj24**

ATGAAATCCTTGAGTGTTTCACTAGTGGTCCTGTGGCTCCAGTTTAATTGGGTGAGCAGCCAGCAGAAGGTGCAGCAGAGCCCAGAATCCCTCACTGTCTCAGAGGGAGCCATGGCCTCCCTCAACTGCACGTTCAGTGATGGTACTTCTAACAACTTCAGGTGGTACAGACAGCATTCTGCGAAAGGCCTTGAGGTGCTAGTGTCCATCTTCTCTGATGGTGAAAAGGAAGAAGGCAGATTCACAGCTCACCTCAATAGAGCCAACTTGCATGTTTCCCTACACATCAGAGAACCACAACCCAGTGACTCTGCTGTCTACCTCTGTGCAGTGAGTCTCACAACTGCCAGTTTGGGGAAACTGCAGTTTGGAACAGGAACCCAGGTTGTGGTGACCCCAGACATCCAGAACCCAGAACCTGCTGTGTACCAGTTAAAAGATCCTCGGTCTCAGGACAGCACCCTCTGCCTGTTCACCGACTTTGACTCCCAAATCAATGTGCCGAAAACCATGGAATCTGGAACGTTCATCACTGACAAAACTGTGCTGGACATGAAAGCTATGGATTCCAAGAGCAATGGGGCCATTGCCTGGAGCAACCAGACAAGCTTCACCTGCCAAGATATCTTCAAAGAGACCAACGCCACCTACCCCAGTTCAGACGTTCCCTGTGATGCCACGTTGACTGAGAAAAGCTTTGAAACAGATATGAACCTAAACTTTCAAAACCTGTCAGTTATGGGACTCCGAATCCTCCTGCTGAAAGTAGCCGGATTTAACCTGCTCATGACGCTGAGGCTGTGGTCCAGTTGA

**EM21 trav16d-dv11 traj26**

ATGCTGATTCTAAGCCTGTTGGGAGCTCCATTTTTTGGCTCCATTTGTTTTGCAACCAGCATGGCCCAGAAGGTAACACAGACTCAGACTTCAATTTCTGTGATGGAGAAGACAACGGTGACAATGGACTGTGTGTATGAAACCCAGGACAGTTCTTACTTCTTATTCTGGTACAAGCAAACAGCAAGTGGGGAAATAGTTTTCCTTATTCGTCAGGACTCTTACAAAAAGGAAAATGCAACAGTGGGTCATTATTCTCTGAACTTTCAGAAGCCAAAAAGTTCCATCGGACTCATCATCACCGCCACACAGATTGAGGACTCGGCAGTATATTTCTGTGCTATGAGAGAGGGCAATAACTATGCCCAGGGATTAACCTTCGGTCTTGGCACCAGAGTATCTGTGTTTCCCTACATCCAGAACCCAGAACCTGCTGTGTACCAGTTAAAAGATCCTCGGTCTCAGGACAGCACCCTCTGCCTGTTCACCGACTTTGACTCCCAAATCAATGTGCCGAAAACCATGGAATCTGGAACGTTCATCACTGACAAAACTGTGCTGGACATGAAAGCTATGGATTCCAAGAGCAATGGGGCCATTGCCTGGAGCAACCAGACAAGCTTCACCTGCCAAGATATCTTCAAAGAGACCAACGCCACCTACCCCAGTTCAGACGTTCCCTGTGATGCCACGTTGACTGAGAAAAGCTTTGAAACAGATATGAACCTAAACTTTCAAAACCTGTCAGTTATGGGACTCCGAATCCTCCTGCTGAAAGTAGCCGGATTTAACCTGCTCATGACGCTGAGGCTGTGGTCCAGTTGA

**EM22 trav6d-4 (not trav6-4 ) traj24**

ATGAATACTTCTCCAGTTTTAGTGACTGCGATGCTGCTGTTCATGCTTGGGATGAGAAAGACCCACGGAGATTCAGTGACCCAGAAACAAGGTCAAGTGACCCTTTCAGAAGATGACTTCCTATTTATAAATTGCACTTATTCTACCACAACGTACCCAACTCTTTTCTGGTATGTCCAATATCCTGGACAAGGTCCACAGCTCCTTCTGAAAGTCACAACTGCCAACAACAAGGGAATCAGCAGAGGCTTTGAAGCTACATATGACAAAGGGACCACCTCCTTCCACTTACAGAAAGCCTCAGTGCAGGAGTCAGACTCAGCCGTGTACTTCTGTGCTCTGGGATTGACAACTGCCAGTTTGGGGAAACTGCAGTTTGGAACAGGAACCCAGGTTGTGGTGACCCCAGACATCCAGAACCCAGAACCTGCTGTGTACCAGTTAAAAGATCCTCGGTCTCAGGACAGCACCCTCTGCCTGTTCACCGACTTTGACTCCCAAATCAATGTGCCGAAAACCATGGAATCTGGAACGTTCATCACTGACAAAACTGTGCTGGACATGAAAGCTATGGATTCCAAGAGCAATGGGGCCATTGCCTGGAGCAACCAGACAAGCTTCACCTGCCAAGATATCTTCAAAGAGACCAACGCCACCTACCCCAGTTCAGACGTTCCCTGTGATGCCACGTTGACTGAGAAAAGCTTTGAAACAGATATGAACCTAAACTTTCAAAACCTGTCAGTTATGGGACTCCGAATCCTCCTGCTGAAAGTAGCCGGATTTAACCTGCTCATGACGCTGAGGCTGTGGTCCAGTTGA

**EM24 trav7-4 (not trav7n-4 ) traj42**

ATGAAATCCTTGAGTGTTTCACTAGTGGTCCTGTGGCTCCAGTTAAACTGCGTGAGGAGCCAGCAGAAGGTGCAGCAGAGCCCAGAATCCCTCAGTGTCCCAGAGGGAGGCATGGCCTCTCTCAACTGCACTTCAAGTGATCGTAATTTTCAGTACTTCTGGTGGTACAGACAGCATTCTGGAGAAGGCCCCAAGGCACTGATGTCAATCTTCTCTGATGGTGACAAGAAAGAAGGCAGATTCACAGCTCACCTCAATAAGGCCAGCCTGCATGTTTCCCTGCACATCAGAGACTCCCAGCCCAGTGACTCCGCTCTCTACTTCTGTGCAGTTAGGAATTCTGGAGGAAGCAATGCAAAGCTAACCTTCGGGAAAGGCACTAAACTCTCTGTTAAATCAAACATCCAGAACCCAGAACCTGCTGTGTACCAGTTAAAAGATCCTCGGTCTCAGGACAGCACCCTCTGCCTGTTCACCGACTTTGACTCCCAAATCAATGTGCCGAAAACCATGGAATCTGGAACGTTCATCACTGACAAAACTGTGCTGGACATGAAAGCTATGGATTCCAAGAGCAATGGGGCCATTGCCTGGAGCAACCAGACAAGCTTCACCTGCCAAGATATCTTCAAAGAGACCAACGCCACCTACCCCAGTTCAGACGTTCCCTGTGATGCCACGTTGACTGAGAAAAGCTTTGAAACAGATATGAACCTAAACTTTCAAAACCTGTCAGTTATGGGACTCCGAATCCTCCTGCTGAAAGTAGCCGGATTTAACCTGCTCATGACGCTGAGGCTGTGGTCCAGTTGA

**EM25 trav7-4 traj31**

ATGAAATCCTTGAGTGTTTCACTAGTGGTCCTGTGGCTCCAGTTAAACTGCGTGAGGAGCCAGCAGAAGGTGCAGCAGAGCCCAGAATCCCTCAGTGTCCCAGAGGGAGGCATGGCCTCTCTCAACTGCACTTCAAGTGATCGTAATTTTCAGTACTTCTGGTGGTACAGACAGCATTCTGGAGAAGGCCCCAAGGCACTGATGTCAATCTTCTCTGATGGTGACAAGAAAGAAGGCAGATTCACAGCTCACCTCAATAAGGCCAGCCTGCATGTTTCCCTGCACATCAGAGACTCCCAGCCCAGTGACTCCGCTCTCTACTTCTGTGCAGCTTTTAATAGCAATAACAGAATCTTCTTTGGTGATGGGACGCAGCTGGTGGTGAAGCCCAACATCCAGAACCCAGAACCTGCTGTGTACCAGTTAAAAGATCCTCGGTCTCAGGACAGCACCCTCTGCCTGTTCACCGACTTTGACTCCCAAATCAATGTGCCGAAAACCATGGAATCTGGAACGTTCATCACTGACAAAACTGTGCTGGACATGAAAGCTATGGATTCCAAGAGCAATGGGGCCATTGCCTGGAGCAACCAGACAAGCTTCACCTGCCAAGATATCTTCAAAGAGACCAACGCCACCTACCCCAGTTCAGACGTTCCCTGTGATGCCACGTTGACTGAGAAAAGCTTTGAAACAGATATGAACCTAAACTTTCAAAACCTGTCAGTTATGGGACTCCGAATCCTCCTGCTGAAAGTAGCCGGATTTAACCTGCTCATGACGCTGAGGCTGTGGTCCAGTTGA
