# Supplementary material for: The Abundance of α-Chain-Centric TCRs in the Mouse Repertoire of Primarily Activated Effectors and Reactivated Memory T Cells
Source: Comput Struct Biotechnol J. 2026 Apr 8;35(1):0026. doi: 10.34133/csbj.0026 (PMC13082541; doi:10.34133/csbj.0026)
Supplement: Supplementary 1 — Figs. S1 and S2 Tables S1 to S3 Supplementary Data 1 and 2 [file csbj.0026.f1.zip › Supplementary Table 1.docx]

**Supplementary Table 1.** TCRα clonotypes of primarily activated effectors (EF) **(A)** and reactivated memory T cells (EM) **(B)** selected for *in vitro* functional testing.

**A)**

| **TCRα** | **CDR3 aa**  **sequence** | **TRAV gene segment** | **Frequency** | | **Pgen* (x10^-4^)** |
| --- | --- | --- | --- | --- | --- |
|  |  |  | **Before stimulation** | **After stimulation** |  |
| EF1 | CAVRTSSGQKLVF | TRAV7DN-6 | 2.23189E-05 | 0.0020 | 0.315 |
| EF2 | CAAETSNYQLIW | TRAV4N-4 | 2.23189E-05 | 0.0018 | 0.004 |
| EF3 | CAADRGSALGRLHF | TRAV14-1 | 2.23189E-05 | 0.0018 | 0.258 |
| EF4 | CAALNSNNRIFF | TRAV1 | 2.23189E-05 | 0.0015 | 0.107 |
| EF5 | CALMDSNYQLIW | TRAV12-3 | 2.23189E-05 | 0.0015 | 0.207 |
| EF6 | CLTEGADRLTF | TRAV12-2 | 2.23189E-05 | 0.0014 | 0.005 |
| EF7 | CALSEASSGSWQLIF | TRAV12D-2 | 2.23189E-05 | 0.0012 | 0.318 |
| EF10 | CAATPNAYKVIF | TRAV14D-2 | 2.23189E-05 | 0.0011 | 0.005 |
| EF11 | CAFRNNYAQGLTF | TRAV13-1 | 2.23189E-05 | 0.0010 | 0.020 |
| EF13 | CAASLYAQGLTF | TRAV10D | 2.23189E-05 | 0.0009 | 0.023 |
| EF14 | CAAGGTNAYKVIF | TRAV14-1 | 2.2838E-05 | 0.0009 | 0.014 |
| EF15 | CVLGMSNYNVLYF | TRAV6-2 | 2.23189E-05 | 0.0009 | 0.090 |
| EF18 | CALSDRGNYAQGLTF | TRAV12N-3 | 2.23189E-05 | 0.0008 | 0.014 |
| EF19 | CALPSSNTNKVVF | TRAV12-2 | 2.23189E-05 | 0.0007 | 0.012 |
| EF21 | CALSDGNYAQGLTF | TRAV12-2 | 2.23189E-05 | 0.0007 | 0.065 |
| EF22 | CAVSATNAYKVIF | TRAV3D-3 | 4.46379E-05 | 0.0013 | 0.195 |
| EF26 | CALPNSGTYQRF | TRAV12-3 | 2.23189E-05 | 0.001 | 0.100 |
| EF27 | CALGDSNYQLIW | TRAV12-3 | 2.23189E-05 | 0.0009 | 0.394 |
| EF29 | CAAASSGSWQLIF | TRAV7-2 | 2.23189E-05 | 0.0007 | 0.760 |
| EF30 | CALSERTGNYKYVF | TRAV12-2 | 2.23189E-05 | 0.0006 | 0.015 |

*****Pgen- probability of CDR3 sequence generation

**B)**

| **TCRα** | **CDR3 aa**  **sequence** | **TRAV gene segment** | **Frequency** | | **Pgen* (x10^-4^)** |
| --- | --- | --- | --- | --- | --- |
|  |  |  | **Before stimulation** | **After stimulation** |  |
| EM1 | CAVRGGNYKYVF | TRAV7-5 | 2.96745E-05 | 0.0010 | 0.019 |
| EM2 | CAASELAGAKLTF | TRAV7-4 | 2.96745E-05 | 0.0008 | 0.001 |
| EM3 | CILRVPPQQGTGSKLSF | TRAV21 | 2.96745E-05 | 0.0008 | 0.001 |
| EM4 | CARNAGAKLTF | TRAV4D-3 | 2.96745E-05 | 0.0008 | 0.053 |
| EM5 | CAMRGTGGYKVVF | TRAV16N | 2.96745E-05 | 0.0007 | 0.174 |
| EM6 | CAVRNSGGSNAKLTF | TRAV7-4 | 2.96745E-05 | 0.0010 | 0.242 |
| EM7 | CALGVSNNRIFF | TRAV6N-7 | 2.96745E-05 | 0.0007 | 0.058 |
| EM8 | CALRGSSGNKLIF | TRAV12N | 2.96745E-05 | 0.0006 | 0.391 |
| EM9 | CAARGSALGRLHF | TRAV4D-4 | 2.96745E-05 | 0.0006 | 0.514 |
| EM10 | CAVRNSAGNKLTF | TRAV7-5 | 2.96745E-05 | 0.0006 | 0.248 |
| EM12 | CVLGDQSSGSWQLIF | TRAV6-2 | 2.96745E-05 | 0.0005 | 0.011 |
| EM13 | CAMRPDNNNAPRF | TRAV8-1 | 5.93489E-05 | 0.0009 | 0.0004 |
| EM15 | CALSGRGSALGRLHF | TRAV12N-3 | 2.96745E-05 | 0.0004 | 0.222 |
| EM17 | CAMRGMDSNYQLIW | TRAV16N | 5.93489E-05 | 0.0007 | 0.020 |
| EM18 | CAMRMTNSAGNKLTF | TRAV16N | 5.93489E-05 | 0.0007 | 0.014 |
| EM19 | CAVSLTTASLGKLQF | TRAV7-5 | 2.96745E-05 | 0.0003 | 0.000004 |
| EM21 | CAMREGNNYAQGLTF | TRAV16D-DV11 | 2.96745E-05 | 0.0003 | 0.118 |
| EM22 | CALGLTTASLGKLQF | TRAV6D-4 | 8.90234E-05 | 0.0008 | 0.000001 |
| EM24 | CAAFNSNNRIFF | TRAV7-4 | 2.96745E-05 | 0.0002 | 0.014 |
| EM25 | CGTNAYKVIF | TRAV7-4 | 2.96745E-05 | 0.0009 | 0.035 |

*****Pgen- probability of CDR3 sequence generation
