# Supplementary material for: The Abundance of α-Chain-Centric TCRs in the Mouse Repertoire of Primarily Activated Effectors and Reactivated Memory T Cells
Source: Comput Struct Biotechnol J. 2026 Apr 8;35(1):0026. doi: 10.34133/csbj.0026 (PMC13082541; doi:10.34133/csbj.0026)
Supplement: Supplementary 1 — Figs. S1 and S2 Tables S1 to S3 Supplementary Data 1 and 2 [file csbj.0026.f1.zip › Supplementary Table 2.docx]

**Supplementary table 2.** Analyses of the functional activity of T cells transduced with individual TCRα originated from TCRs of primarily effectors (EF) **(A)** or reactivated memory T cells (EM) **(B)**

**A)**

| **TCRα** | **Functional test** | | |
| --- | --- | --- | --- |
|  | **MLR** | **MLTC** | **CTL-test** |
| EF1 | **+** | - | + |
| EF2 | **+** | - | + |
| EF3 | **+** | + | + |
| EF4 | - | - | x |
| EF5 | - | - | x |
| EF6 | - | - | x |
| EF7 | - | - | x |
| EF10 | - | - | x |
| EF11 | - | - | x |
| EF13 | + | - | + |
| EF14 | - | - | x |
| EF15 | - | - | х |
| EF18 | - | - | x |
| EF19 | - | - | x |
| EF21 | - | - | x |
| EF22 | - | - | x |
| EF26 | - | - | x |
| EF27 | - | - | - |
| EF29 | - | - | - |
| EF30 | - | - | - |

“+” – functionally active; “-“ – functionally not active; “x” - not done

**B)**

| **TCRα** | **Functional test** | | |
| --- | --- | --- | --- |
|  | **MLR** | **MLTC** | **CTL-test** |
| EM1 | - | - | х |
| EM2 | - | + | + |
| EM3 | - | - | x |
| EM4 | - | - | x |
| EM5 | + | + | + |
| EM6 | - | - | x |
| EM7 | - | - | х |
| EM8 | + | - | + |
| EM9 | + | - | + |
| EM10 | + | + | + |
| EM12 | - | - | x |
| EM13 | - | - | x |
| EM15 | - | - | x |
| EM17 | - | - | x |
| EM18 | - | - | х |
| EM19 | - | - | х |
| EM21 | - | - | х |
| EM22 | - | - | х |
| EM24 | - | - | х |
| EM25 | - | - | х |

“+” – functionally active; “-“ – functionally not active; “x”- not done
