# Supplementary material for: The Abundance of α-Chain-Centric TCRs in the Mouse Repertoire of Primarily Activated Effectors and Reactivated Memory T Cells
Source: Comput Struct Biotechnol J. 2026 Apr 8;35(1):0026. doi: 10.34133/csbj.0026 (PMC13082541; doi:10.34133/csbj.0026)
Supplement: Supplementary 1 — Figs. S1 and S2 Tables S1 to S3 Supplementary Data 1 and 2 [file csbj.0026.f1.zip › Supplementary Table 3.docx]

**Supplementary table 3.** Vα families of identified α-chain-centric TCR clonotypes.

| **α-chain-centric TCR** | **TRAV segment** | **Vα family** |
| --- | --- | --- |
| **P815 tumor model (this study)** | | |
| EF1 | TRAV7DN-6 | Vα1 |
| EF2 | TRAV4N-4 | Vα11 |
| EF3 | TRAV14-1 | Vα2.2 |
| EF13 | TRAV10D | Vα22.2 |
| EM2 | TRAV7-4 | Vα1 |
| EM5 | TRAV16N | Vα16 |
| EM8 | TRAV12N | Vα8 |
| EM9 | TRAV4D-4 | Vα11 |
| EM10 | TRAV7-5 | Vα1 |
| **EL-4 tumor model [17]** | | |
| 1D1a | TRAV4D-3 | Vα11.3 |
| ***S. typhimurium* infection model [2]** | | |
| SM1 | TRAV14D-1 | Vα2 |
| SM14 | TRAV9-1 | Vα3 |
| SM16 | TRAV16N | Vα16 |
| SM20 | TRAV3-1 | Vα5 |
| SM21 | TRAV16N | Vα16 |
