# Supplementary material for: The Abundance of α-Chain-Centric TCRs in the Mouse Repertoire of Primarily Activated Effectors and Reactivated Memory T Cells
Source: Comput Struct Biotechnol J. 2026 Apr 8;35(1):0026. doi: 10.34133/csbj.0026 (PMC13082541; doi:10.34133/csbj.0026)
Supplement: Supplementary 1 — Figs. S1 and S2 Tables S1 to S3 Supplementary Data 1 and 2 [file csbj.0026.f1.zip › Supplementary Figure legends.docx]

**Supplementary Figure legends**

**Supplementary Figure 1. CDR3 physicochemical parameters of α-chain TCRs selected for cloning and *in vitro* tests.** The length **(A)**, volume **(B)**, hydropathy **(C)**, charge **(D)**, polarity **(E)**, and strength **(F)** were calculated for the selected α-chain TCRs in the repertoire of primarily activated effectors (EF) and reactivated memory cells (EM) and compared with the values of the TOP-100 TCRα clonotypes in the respective repertoire. Unpaired Student’s t-test.

**Supplementary Figure 2. T cells transduced with the dominant-active α-chains didn’t kill syngeneic lymphoma EL-4.** T cells from intact C57BL/6 mice (H-2^b^) were modified with dominant-active TCRα from the repertoire of primarily activated effectors (EF) or reactivated memory T cells (EM), identified in MLR and MLTC screening tests (Fig. 1, Fig. 2). Transduced T cells were mixed with the syngeneic lymphoma EL-4-GFP (H-2K^b^) at a ratio of 2:1. Non-transduced (NTR) T cells were used as the control. Cytotoxic activity of transduced T cells was analyzed by flow cytometry after 24 h of culturing by evaluating the percentage of dead EL-4 tumor cells as GFP^+^PI^+^ cells. Data from 2-4 independent experiments are shown as mean ± SD.
