# Supplementary figures and images for: The Abundance of α-Chain-Centric TCRs in the Mouse Repertoire of Primarily Activated Effectors and Reactivated Memory T Cells
Source: Comput Struct Biotechnol J. 2026 Apr 8;35(1):0026. doi: 10.34133/csbj.0026 (PMC13082541; doi:10.34133/csbj.0026)

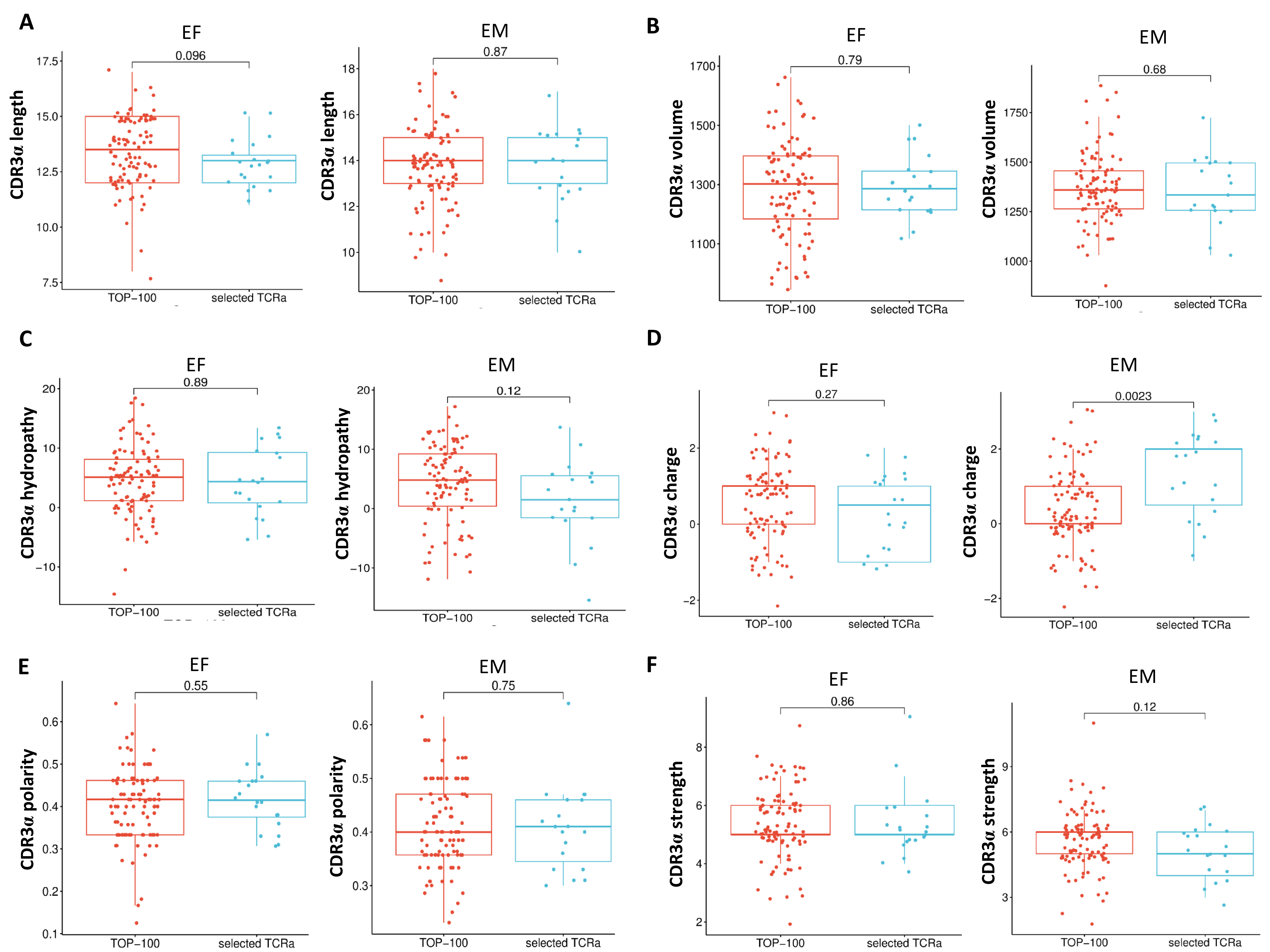

Supplement: Supplementary 1 — Figs. S1 and S2 Tables S1 to S3 Supplementary Data 1 and 2 [file csbj.0026.f1.zip › Supplementary Fig. 1.tiff]

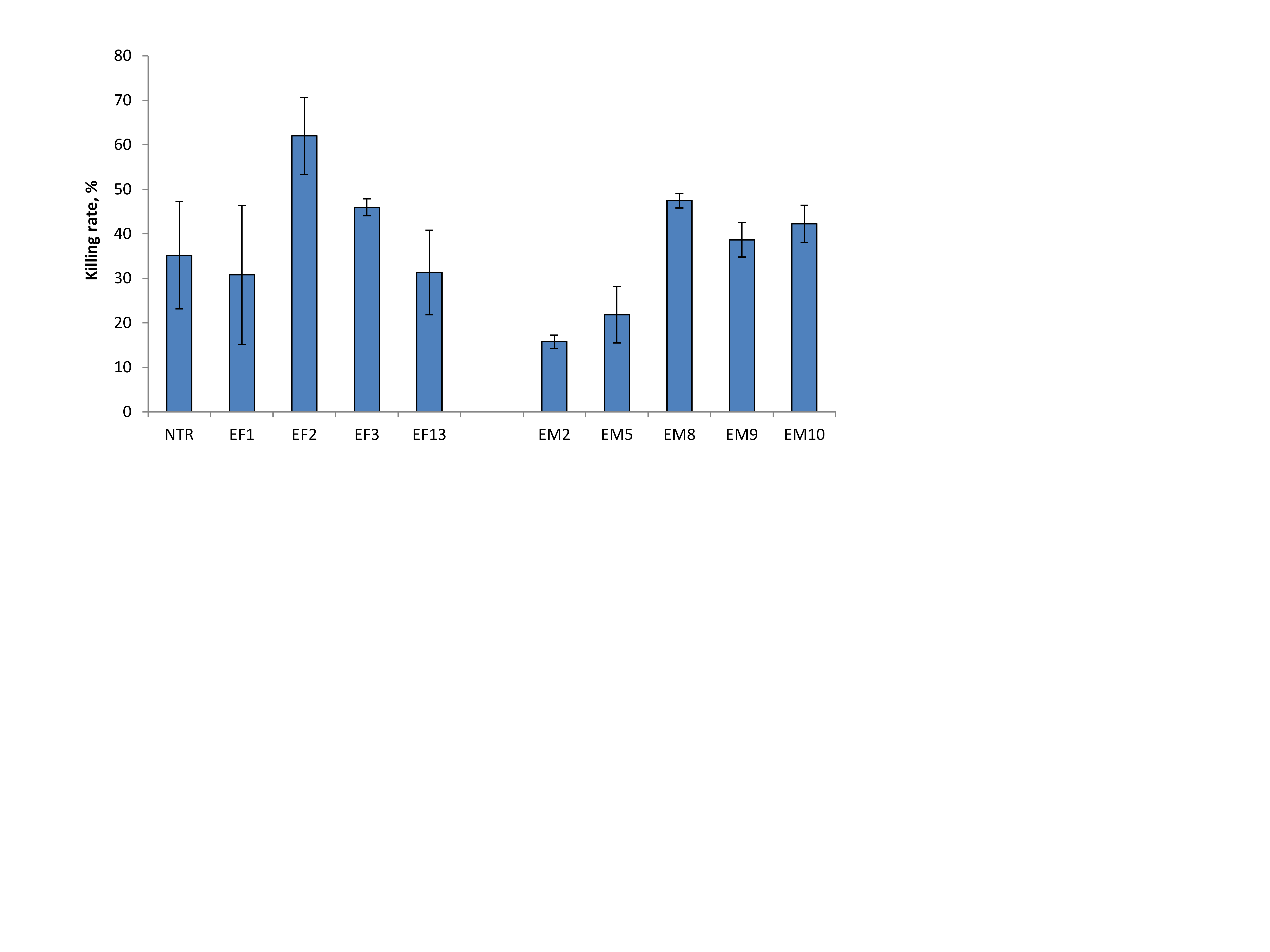

Supplement: Supplementary 1 — Figs. S1 and S2 Tables S1 to S3 Supplementary Data 1 and 2 [file csbj.0026.f1.zip › Supplementary Fig. 2.tiff]
